# Meningococcal serogroups and surveillance: a systematic review and survey

**DOI:** 10.7189/jogh.09.010409

**Published:** 2019-06

**Authors:** Meagan E Peterson, You Li, André Bita, Annick Moureau, Harish Nair, Moe H Kyaw, Meningococcal Surveillance Group (in alphabetical order), Raquel Abad, Freddie Bailey, Isabel de la Fuente Garcia, Antoaneta Decheva, Pavla Krizova, Tanya Melillo, Anna Skoczynska, Nadezhda Vladimirova

**Affiliations:** 1 Centre for Global Health Research, Usher Institute of Population Health Sciences and Informatics, University of Edinburgh, Edinburgh, Scotland, UK; 2 World Health Organization: Inter-Country Support Team for West Africa, Ouagadougou, Burkina Faso; 3 Sanofi Pasteur, Marcy l’Etoile, France; 4 Public Health Foundation of India, New Delhi, India; 5 Sanofi Pasteur, Swiftwater, Pennsylvania, USA; 6 National Centre for Microbiology, Instituto de Salud Carlos III, Madrid, Spain; 7 Medical School, University of Edinburgh, Edinburgh, Scotland, UK; 8 Kannerklinik, Centre Hospitalier du Luxembourg, Luxembourg; 9 National Center of Infectious and Parasitic Diseases, Sofia, Bulgaria; 10 National Reference Laboratory for Meningococcal Infections, National Institute of Public Health, Prague, Czech Republic; 11 Infectious Disease Prevention and Control Unit, Msida, Malta; 12 National Reference Centre for Bacterial Meningitis, National Medicines Institute, Warsaw, Poland; * Joint authors in this position

## Abstract

**Background:**

Meningococcal disease continues to be a global public health concern due to its epidemic potential, severity, and sequelae. The global epidemiological data on circulating meningococcal serogroups have never been reviewed concurrently with the laboratory capacity for meningococcal surveillance at the national level. We, therefore, aimed to conduct a country-level review of meningococcal surveillance, serogroup distribution, and vaccine use.

**Methods:**

We conducted a systematic literature review across six databases to identify studies (published January 1, 2010 to October 16, 2017) and grey literature reporting meningococcal serogroup data for the years 2010-2016. We performed independent random effects meta-analyses for serogroups A, B, C, W, X, Y, and other. We developed and circulated a questionnaire-based survey to surveillance focal points in countries (N = 95) with known regional bacterial meningitis surveillance programs to assess their surveillance capacity and summarized using descriptive methods.

**Results:**

We included 173 studies from 59 countries in the final analysis. The distribution of meningococcal serogroups differed markedly between countries and regions. Meningococcal serogroups C and W accounted for substantial proportions of meningococcal disease in most of Africa and Latin America. Serogroup B was the predominant cause of meningococcal disease in many locations in Europe, the Americas, and the Western Pacific. Serogroup Y also caused many cases of meningococcal disease in these regions, particularly in Nordic countries. Survey responses were received from 51 countries. All countries reported the ability to confirm the pathogen in-country, while approximately 30% either relied on reference laboratories for serogrouping (N = 10) or did not serogroup specimens (N = 5). Approximately half of countries did not utilize active laboratory-based surveillance system (N = 22). Nationwide use of a meningococcal vaccine varied, but most countries (N = 36) utilized a meningococcal vaccine at least for certain high-risk population groups, in private care, or during outbreaks.

**Conclusions:**

Due to the large geographical variations in circulating meningococcal serogroups, each country should continue to be monitored for changes in major disease-causing serogroups in order to inform vaccine and control policies. Similarly, laboratory capacity should be appropriately scaled up to more accurately understand local epidemiology and disease burden, as well as the impact of vaccination programs.

Invasive meningococcal disease (IMD), caused by the bacteria *Neisseria meningitidis*, most commonly manifests as meningitis or septicemia [1]. Historically, more than 80% of cases were fatal [2,3]. Despite the availability of effective antibiotics, IMD is still associated with a case fatality rate of approximately 10%-15% [4,5], which can increase to 40% for meningococcemia cases during outbreaks [5,6]. Approximately 10%-20% of survivors have severe neurological, visual, or hearing impairments, rates of which are often higher in low-resource settings [5,7,8]. Approximately 1.2 million cases of IMD occur each year, resulting in about 335 000 deaths worldwide [1]. The burden of IMD is disproportionately higher in the African meningitis belt which has had the greatest number of meningococcal epidemics [9]. IMD incidence is highest among infants and children under 5, with a second peak in incidence among adolescents [9]. Additionally, IMD outbreaks have been reported among university students, military recruits, and Hajj pilgrims [10-13].

Virulent *Neisseria meningitidis* is predominantly encapsulated, and of the 12 identified capsular serogroups, A (NmA), B (NmB), C (NmC), W (NmW), X (NmX), and Y (NmY) cause the vast majority of IMD cases [14]. The dynamic epidemiology of these serogroups is unpredictable and varies with time and geographical region [15]. Knowledge of the local serogroup prevalence is gained through adequate surveillance activities within a country. Additionally, surveillance provides data on disease burden and outbreak detection. Together, this information guides vaccine and prevention policies [16].

Immunization against IMD is the best prevention method. Currently, there are vaccines available for all major disease-causing serogroups (A, B, C, W, and Y) except serogroup X, which has multiple versions in development [17,18]. Although, it should be noted that the vaccine targeting serogroup B is only broadly protective and does not cover all strains causing disease [19]. Meningococcal vaccines are serogroup specific, or protein-specific in the case of the vaccines targeting serogroup B [19], thus further necessitating the knowledge of the circulating serogroups within a country. Although surveillance capacity is needed to determine the direct and indirect effects of existing vaccines and any newly introduced vaccines [15,16], many countries either do not have laboratory-based meningococcal surveillance systems or have limited participation in surveillance activities [15,20-22]. The development of effective vaccine policies and determination of suitable vaccines for use in a country is dependent upon the local serogroup epidemiology and cost-effectiveness. Although laboratory capacity, serogroup distribution, and vaccine use have been reviewed for select countries prior to 2010 [23-28], to our knowledge, no recent article has provided the comprehensive data on these three aspects globally for the post-2010 period. Since these topics are interrelated, we aimed to conduct a country-level review of meningococcal surveillance through the investigation of current serogroup distribution, global laboratory capacity for meningococcal surveillance, and vaccine use within countries.

## METHODS

### Search strategy and data sources

We conducted a systematic literature review to identify published studies reporting meningococcal serogroup data according to a pre-specified protocol (PROSPERO number CRD42017080219). We identified articles published from January 1, 2010 to October 16, 2017 that reported country specific invasive meningococcal disease serogroup data for 2010-2016 from 6 databases: MEDLINE, Embase, Web of Science Core Collection, Current Contents Connect, WHO Global Health Library, and Global Health Database. Detailed search strategy including search terms can be found in the Supplementary material. No restriction was made based upon language. Where required, we used Google Translate to assist in translation of non-English language articles. Additionally, we sought assistance of a native speaker and/or contacted authors to seek clarifications. We also identified unpublished national surveillance data by searching relevant ministry of health and surveillance network websites for each country.

### Inclusion and exclusion criteria

Studies or surveillance reports were included if they: reported meningococcal serogroup data from typically sterile sites in humans; clearly specified the number of samples tested and the serogroups identified; clearly described the time period and country of specimen collection; reported the majority of data between January 1, 2010 and December 31, 2016; and had a sample size of at least 15 (or an average of 15 per year if reporting for more than one year). Studies in all age groups were eligible for inclusion. Studies from outbreak periods were eligible for inclusion. Studies were excluded if they were conducted only in a select population (ie, patients with asplenia) or only tested for one serogroup.

### Data collection and management

Two authors (MEP and YL) independently reviewed full text articles and extracted relevant data into a database in Microsoft Access. Any differences in extraction were resolved through discussion. Only one study was chosen for each geographic area and time period. If multiple studies were identified for the same location and time period, the choice between studies was based upon quality, representativeness of geographic area, number of serogroups reported, recent study year, and sample size. We contacted authors of identified publications for any relevant additional information. For several countries in the meningitis belt, multiple studies were identified where the risk of duplicate data could not be reconciled. The two primary reasons included: multiple studies stating the data are reported from the same sources, yet the studies provide different data; and uncertainty about the representativeness of the studies. In these instances, the study that was deemed to be most representative with the largest sample size was included in the analysis, and the remaining are detailed in Table S1 in **Online Supplementary Document**. Questions concerning appropriateness for inclusion in analysis were discussed between MEP, YL, HN, and MHK and agreed upon prior to proceeding to analysis.

### Data analysis

We conducted meta-analyses per serogroup: NmA, NmB, NmC, NmW, NmX, NmY, and Other Nm (which includes non-groupable, non-encapsulated, NmE, NmE/Z, NmZ, polyagglutinable, and incompletely identified serogroups). All reported serogroups were included in the calculation of the denominator, while unknown or untested samples were excluded. In cases where we could be reasonably certain that a serogroup was tested for, but simply not reported because there were zero cases, we included an assumed zero for that serogroup (Table S3 in the **Online Supplementary Document**). If a study reported serogroup data by year, each year was included as a separate datapoint in the analysis. In order to calculate a point estimate for each country, a random effects model using exact binomial confidence intervals and the Freeman-Tukey transformation were utilized. All analyses were performed in StataIC 13 (64-bit) with the *metaprop* command [29]. Results are organized by WHO Regions.

### Survey design and distribution

We developed and piloted a questionnaire (Table S2 in **Online Supplementary Document**) with specific reference to general meningococcal surveillance and laboratory capacity for surveillance. After drafting the survey, we circulated it to several content experts who reviewed it for content validity and revised it according to their suggestions. We then contacted WHO Regional Office staff involved with the Global Invasive Bacterial Vaccine Preventable Diseases (IB-VPD) Surveillance Network (GISN) and European Centre for Disease Prevention and Control (ECDC) officers and sought their assistance to invite national Ministry of Health or Surveillance Programme officers to participate in this exercise. The questionnaire was sent to the regional contacts between October 25, 2016 and November 11, 2016, and reminders were sent as part of regular follow up if survey was not received.

### Questionnaire analysis

Completed questionnaires were extracted into Microsoft Access by MEP and independently crosschecked by YL. If upon review of the responses clarifications were needed, questions were sent to the country-level contact for more detail. The data from the questionnaires were synthesized using descriptive methods. We used WHO Regional classification to compare laboratory capacity for surveillance at the regional level.

## RESULTS

**Figure 1:**
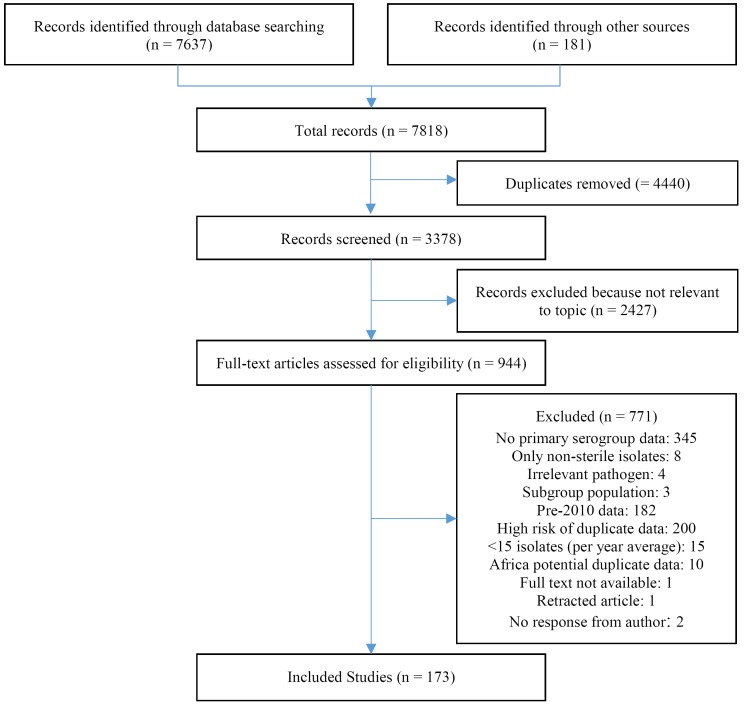
PRISMA flowchart of included studies.

We identified a total of 7637 articles through literature review and an additional 181 through hand searching. We reviewed 994 articles in full and identified 173 articles meeting our strict eligibility criteria and reporting relevant data (**Figure 1**). We included data from 59 countries, representing, at least partially, all six WHO regions. Study characteristics can be found in Table S3 in the **Online Supplementary Document**. Data availability differed substantially between WHO regions (Table S3 in **Online Supplementary Document**). The regions with greatest representation included the European (EURO), African (AFRO), and the Americas (AMRO) with 51% (27/53), 36% (17/47), and 26% (9/35) of countries with data included, respectively. Within AFRO, substantially more data were available for the meningitis belt (60%, 15/25) compared to non-meningitis belt (9%, 2/22). The remaining three WHO regions had substantially less representation. Data from only 15% (4/27) of the Western Pacific (WPRO), 10% (2/21) of the Eastern Mediterranean (EMRO), and 9% (1/11) South-East Asian (SEARO) were included in the analysis. Results of country-level meta-analyses (by WHO region) are presented in **Table 1****.**

**Table 1:** Estimated percent (%) of invasive meningococcal disease cases caused by the respective serogroup in each country (organized by WHO region)*

African Region (AFRO)							
Country (years)	**NmA (95% CI)**	**NmB (95% CI)**	**NmC (95% CI)**	**NmW (95% CI)**	**NmX (95% CI)**	**NmY (95% CI)**	**Other Nm (95% CI)**
Algeria (2010, 2011, 2015)	10.8 (4.1-19.7)	59.3 (47.3-70.7)	6.4 (0.0-19.4)	11.9 (3.1-24.3)	–	4.2 (0.3-10.9)	4.0 (0.0-12.3)
Benin (2012)	–	–	–	84.5 (74.0-92.0)	15.5 (8.0-26.0)	–	–
Burkina Faso (2010-2016)	0.7 (0.0-2.7)	0.0 (0.0-0.4)	2.3 (0.0-9.1)	72.1 (42.7-93.7)	22.5 (5.1-47.4)	0.1 (0.0-0.3)	–
Cameroon (2010-2012)	76.2 (45.8-96.8)	0.0 (0.0-1.0)	0.0 (0.0-1.0)	23.8 (3.2-54.2)	0.0 (0.0-1.0)	0.0 (0.0-1.0)	–
Central African Republic (2016)	1.9 (0.1-9.9)	0.0 (0.0-6.6)	0.0 (0.0-6.6)	98.2 (90.1-100.0)	0.0 (0.0-6.6)	0.0 (0.0-6.6)	–
Chad (2010-2012)	90.3 (75.5-98.9)	0.0 (0.0-5.8)	0.0 (0.0-5.8)	7.8 (0.1-23.7)	1.1 (0.0-3.0)	0.0 (0.0-5.8)	–
Cote d'Ivoire (2012)	0.0 (0.0-4.0)	1.1 (0.0-6.0)	1.1 (0.0-6.0)	97.8 (92.2-99.7)	0.0 (0.0-4.0)	0.0 (0.0-4.0)	–
Ethiopia (2015)	12.5 (1.6-38.4)	6.3 (0.0-30.2)	31.3 (11.0-58.7)	50.0 (24.7-75.4)	0.0 (0.0-20.6)	0.0 (0.0-20.6)	–
The Gambia (2012)	3.6 (0.1-18.4)	0.0 (0.0-12.3)	0.0 (0.0-12.3)	96.4 (81.7-99.9)	0.0 (0.0-12.3)	0.0 (0.0-12.3)	–
Ghana (2010, 2012-2016)	1.9 (0.0-6.8)	1.1 (0.0-5.0)	1.7 (0.1-4.4)	91.2 (86.6-95.0)	0.0 (0.0-0.2)	1.1 (0.1-2.9)	–
Guinea (2013, 2015)	97.3 (92.0-100.0)	0.0 (0.0-1.4)	0.0 (0.0-1.4)	2.8 (0.0-8.0)	0.0 (0.0-1.4)	0.0 (0.0-1.4)	–
Mali (2010-2012, 2015, 2016)	13.0 (0.0-62.4)	0.0 (0.0-0.8)	7.4 (0.0-28.4)	48.1 (0.6-98.4)	11.7 (0.0-41.8)	0.0 (0.0-0.8)	–
Niger (2010-2012, 2014-2016)	1.6 (0.0-13.3)	0.0 (0.0-<0.1)	25.1 (0.0-78.9)	63.2 (24.4-94.1)	0.2 (0.0-1.4)	0.0 (0.0-<0.1)	1.0 (0.5-1.6)
Nigeria (2010, 2014, 2015)	8.0 (0.0-48.9)	0.0 (0.0-3.1)	100.0 (96.9-100.0)	11.0 (0.0-65.0)	0.0 (0.0-3.1)	0.0 (0.0-3.1)	–
Senegal (2012)	18.2 (5.2-40.3)	0.0 (0.0-15.4)	0.0 (0.0-15.4)	81.8 (59.7-94.8)	0.0 (0.0-15.4)	0.0 (0.0-15.4)	–
South Africa (2010-2015)	0.2 (0.0-0.7)	29.1 (25.4-33.0)	9.4 (7.9-11.1)	43.9 (38.6-49.3)	0.2 (0.0-0.6)	15.3 (13.3-17.4)	0.9 (0.2-1.9)
Togo (2015, 2016)	0.2 (0.0-1.3)	0.0 (0.0-0.2)	0.0 (0.0-0.2)	99.8 (98.7-100.0)	0.0 (0.0-0.2)	0.0 (0.0-0.2)	–
Region of The Americas (AMRO)							
Country	**NmA (95% CI)**	**NmB (95% CI)**	**NmC (95% CI)**	**NmW (95% CI)**	**NmX (95% CI)**	**NmY (95% CI)**	**Other Nm (95% CI)**
Argentina (2010-2016)	0.0 (0.0-0.2)	44.7 (40.7-48.8)	4.1 (2.5-6.2)	46.5 (41.1-51.9)	0.1 (0.0-0.5)	3.1 (2.0-4.4)	0.3 (0.0-0.9)
Brazil (2010-2015)	0.0 (0.0-0.1)	21.2 (18.3-24.2)	67.7 (63.2-72.0)	6.4 (5.3-7.5)	0.0 (0.0-0.1)	3.7 (2.6-5.1)	0.3 (0.1-0.5)
Canada (2010, 2011, 2013-2015)	0.0 (0.0-0.3)	63.1 (59.2-67.0)	5.1 (2.6-8.3)	5.8 (4.0-7.9)	0.1 (0.0-0.6)	23.6 (20.0-27.3)	1.8 (0.8-3.1)
Chile (2010-2016)	0.0 (0.0-0.3)	37.6 (28.7-47.0)	3.3 (1.0-6.6)	51.7 (37.3-65.9)	0.0 (0.0-0.3)	1.4 (0.3-3.1)	2.1 (0.6-4.2)
Colombia (2010-2014)	0.0 (0.0-1.4)	67.4 (59.4-75.0)	16.2 (10.1-23.4)	0.1 (0.0-2.1)	0.1 (0.0-2.2)	13.1 (7.3-20.1)	0.2 (0.0-2.4)
Mexico (2010)	0.0 (0.0-19.5)	11.8 (1.5-36.4)	88.2 (63.6-98.5)	0.0 (0.0-19.5)	0.0 (0.0-19.5)	0.0 (0.0-19.5)	0.0 (0.0-19.5)
United States of America (2010-2015)	0.2 (0.0-1.2)	30.8 (24.8-37.1)	23.3 (17.4-29.7)	9.6 (2.5-20.2)	–	25.6 (14.5-38.5)	5.4 (2.8-8.8)
Uruguay (2010-2012)	0.0 (0.0-3.0)	73.0 (60.2-84.2)	7.2 (1.7-15.1)	11.1 (4.2-20.2)	0.0 (0.0-3.0)	4.3 (0.0-13.5)	1.4 (0.0-8.8)
Venezuela (2010-2014)	0.0 (0.0-2.9)	34.6 (25.8-44.0)	52.8 (39.3-66.1)	0.3 (0.0-3.2)	0.0 (0.0-1.9)	8.2 (2.1-16.9)	1.0 (0.0-4.7)
Eastern Mediterranean Region (EMRO)							
Country	**NmA (95% CI)**	**NmB (95% CI)**	**NmC (95% CI)**	**NmW (95% CI)**	**NmX (95% CI)**	**NmY (95% CI)**	**Other Nm (95% CI)**
Morocco (2011-2016)	–	95.8 (91.1-98.4)	2.1 (0.4-6.0)	0.0 (0.0-19.5)	–	1.4 (0.2-5.0)	–
Sudan (2012)	90.0 (68.3-98.8)	0.0 (0.0-16.8)	0.0 (0.0-16.8)	10.0 (1.2-31.7)	0.0 (0.0-16.8)	0.0 (0.0-16.8)	–
European Region (EURO)							
Country	**NmA (95% CI)**	**NmB (95% CI)**	**NmC (95% CI)**	**NmW (95% CI)**	**NmX (95% CI)**	**NmY (95% CI)**	**Other Nm (95% CI)**
Austria (2010-2016)	0.0 (0.0-0.9)	62.7 (57.4-67.9)	22.9 (16.0-30.5)	3.6 (1.6-6.1)	0.0 (0.0-0.6)	6.2 (3.7-9.3)	0.6 (0.0-2.0)
Belgium (2010-2015)	0.0 (0.0-0.4)	73.9 (70.0-77.6)	11.7 (9.3-14.3)	2.8 (1.1-5.2)	0.3 (0.0-1.0)	7.8 (5.3-10.8)	2.7 (1.5-4.1)
Croatia (2010, 2012, 2013)	–	91.7 (73.0-99.0)	4.2 (0.1-21.1)	4.2 (0.1-21.1)	–	3.2 (0.0-11.0)	0.0 (0.0-14.3)
Czech Republic (2010-2016)	0.1 (0.0-1.1)	74.7 (68.0-80.8)	16.4 (11.8-21.7)	3.1 (0.7-6.5)	0.2 (0.0-1.4)	3.4 (1.5-5.8)	2.2 (0.1-11.5)
Denmark (2010-2016)	0.0 (0.0-0.6)	48.4 (40.1-56.8)	30.3 (15.5-47.5)	3.7 (0.2-10.1)	0.1 (0.0-1.2)	8.4 (4.5-13.2)	0.1 (0.0-1.2)
Finland (2010-2016)	0.0 (0.0-1.3)	47.6 (39.9-55.4)	16.5 (11.0-22.8)	5.4 (1.4-11.0)	–	28.4 (21.6-35.7)	–
France (2010-2016)	0.0 (0.0-0.1)	62.2 (55.0-69.2)	22.4 (18.4-26.8)	5.1 (3.3-7.2)	0.3 (0.1-0.5)	8.9 (6.7-11.1)	0.5 (0.2-0.8)
Germany (2010-2016)	0.4 (0.0-0.9)	68.3 (64.4-72.0)	19.8 (17.6-22.1)	4.0 (2.6-5.7)	0.0 (0.0-0.1)	6.9 (5.3-8.8)	0.1 (0.0-0.4)
Greece (2010-2016)	1.1 (0.1-2.8)	84.7 (78.6-90.0)	2.2 (0.1-6.0)	1.2 (0.1-3.0)	0.1 (0.0-1.1)	2.5 (0.6-5.3)	5.1 (2.8-7.9)
Hungary (2010-2015)	0.1 (0.0-1.4)	58.0 (44.2-71.1)	36.0 (23.8-49.1)	0.0 (0.0-0.8)	0.0 (0.0-1.0)	0.7 (0.0-3.1)	1.7 (0.0-7.9)
Ireland (2010-2015)	–	90.1 (81.1-96.6)	4.1 (0.9-8.8)	1.7 (0.4-3.7)	–	2.5 (0.6-5.6)	0.3 (0.0-1.2)
Italy (2010-2016)	0.3 (0.0-0.8)	49.8 (40.0-59.6)	30.6 (22.8-39.0)	4.3 (2.7-6.3)	0.1 (0.0-0.6)	13.9 (11.7-16.3)	0.0 (0.0-3.3)
Kyrgyzstan (2014, 2015)	89.6 (80.9-96.1)	5.4 (0.8-12.5)	4.6 (0.5-11.4)	–	–	–	–
Lithuania (2010-2015)	0.0 (0.0-0.6)	71.9 (61.6-81.1)	4.6 (2.2-7.6)	0.1 (0.0-1.3)	0.0 (0.0-0.7)	0.0 (0.0-0.6)	17.6 (6.3-32.5)
Netherlands (2010-2016)	0.0 (0.0-0.3)	73.4 (64.5-81.5)	4.4 (3.0-6.1)	6.5 (1.1-15.4)	0.2 (0.0-0.9)	11.8 (9.5-14.4)	0.7 (0.1-1.7)
Norway (2010-2016)	0.0 (0.0-9.0)	36.6 (29.5-44.0)	14.6 (5.8-26.0)	4.6 (0.4-11.4)	0.0 (0.0-9.0)	37.3 (28.6-46.3)	0.4 (0.0-2.7)
Poland (2010-2016)	0.0 (0.0-0.4)	62.8 (57.5-68.0)	34.0 (28.0-40.2)	1.6 (0.7-2.7)	0.0 (0.0-0.6)	1.4 (0.8-2.2)	0.0 (0.0-0.4)
Portugal (2010-2015)	0.2 (0.0-1.3)	81.6 (77.3-85.7)	5.4 (3.1-8.2)	0.3 (0.0-1.4)	0.0 (0.0-6.0)	7.0 (2.5-13.1)	3.0 (0.9-5.9)
Romania (2010-2015)	4.9 (1.9-8.9)	68.7 (61.7-75.4)	21.6 (15.7-28.0)	0.9 (0.0-3.7)	0.0 (0.0-1.8)	0.3 (0.0-2.5)	2.0 (0.0-6.5)
Russian Federation (2011, 2014, 2016)	22.0 (14.3-30.7)	40.7 (36.9-44.5)	30.1 (27.2-33.2)	2.6 (1.6-3.8)	0.2 (0.0-1.1)	0.9 (0.3-1.7)	0.6 (0.1-1.7)
Slovakia (2010-2012, 2015, 2016)	0.0 (0.0-2.6)	72.3 (63.6-80.3)	24.3 (16.7-32.7)	1.1 (0.0-4.6)	0.0 (0.0-3.9)	0.8 (0.0-4.1)	0.0 (0.0-2.6)
Spain (2009-2016)	0.3 (0.1-0.6)	72.2 (68.3-75.9)	12.1 (9.5-15.0)	2.9 (1.7-4.4)	–	1.9 (1.0-3.0)	7.0 (5.3-8.8)
Sweden (2010-2016)	0.0 (0.0-0.5)	22.5 (18.7-26.5)	24.2 (17.5-31.5)	8.5 (2.8-16.4)	–	42.4 (35.8-49.1)	0.1 (0.0-1.0)
Switzerland (2010-2015)	0.1 (0.0-1.3)	43.8 (37.1-50.6)	21.2 (15.7-27.3)	10.0 (2.6-20.8)	0.9 (0.0-3.1)	20.5 (15.6-25.9)	0.5 (0.0-2.5)
Turkey (2011-2014)	3.9 (0.9-8.4)	21.0 (4.6-44.0)	0.0 (0.0-1.3)	47.2 (37.0-57.5)	–	0.8 (0.0-3.7)	24.8 (11.4-40.9)
United Kingdom (2009-2016)	0.0 (0.0-0.1)	74.0 (68.1-80.6)	3.1 (2.2-4.2)	9.4 (5.7-13.9)	0.0 (0.0-<0.1)	9.5 (8.1-11.1)	0.1 (0.0-0.5)
South-East Asian Region (SEARO)							
Country	**NmA (95% CI)**	**NmB (95% CI)**	**NmC (95% CI)**	**NmW (95% CI)**	**NmX (95% CI)**	**NmY (95% CI)**	**Other Nm (95% CI)**
India (2010)	100.0 (94.9-100.0)	–	0.0 (0.0-5.1)	–	–	–	0.0 (0.0-5.1)
Western Pacific Region (WPRO)							
Country	**NmA (95% CI)**	**NmB (95% CI)**	**NmC (95% CI)**	**NmW (95% CI)**	**NmX (95% CI)**	**NmY (95% CI)**	**Other Nm (95% CI)**
Australia (2010-2016)	0.0 (0.0-0.2)	72.5 (61.0-82.7)	3.6 (2.0-5.6)	11.9 (4.3-22.5)	–	8.6 (6.1-11.5)	1.8 (0.6-3.5)
China (2010-2014)	12.5 (6.2-20.4)	16.1 (10.1-23.1)	40.3 (30.7-50.2)	7.2 (3.4-12.0)	0.1 (0.0-1.1)	0.0 (0.0-0.7)	21.2 (14.4-28.8)
Japan (2013, 2014)	–	9.8 (2.7-23.1)	17.1 (7.2-32.1)	4.9 (0.6-16.5)	–	61.0 (44.5-75.8)	7.3 (1.5-19.9)
New Zealand (2010-2016)	0.0 (0.0-0.5)	63.3 (58.2-68.2)	22.7 (15.6-30.8)	4.0 (1.2-8.1)	0.0 (0.0-0.5)	6.1 (3.8-8.8)	0.7 (0.0-1.9)

NmA – *Neisseria meningitides* A, NmB – *Neisseria meningitides* B, NmC – *Neisseria meningitides* C, NmW – *Neisseria meningitides* W, NmX – *Neisseria meningitides* X, NmY – *Neisseria meningitides* Y, Nm – Neisseria meningitidis, CI – confidence interval

*Since independent meta-analyses were conducted, numbers may not sum to 100%.

**Figure 2:**
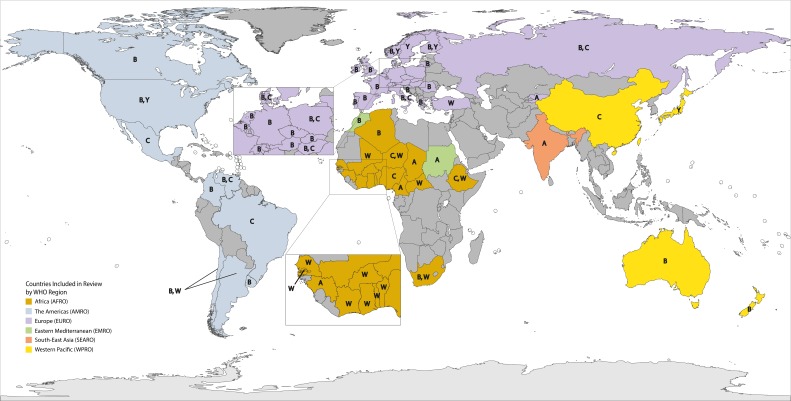
Countries included in serogroup review. Serogroups shown represent an estimated ≥25% of cases for 2010-2016.

We observed substantial differences in the distribution of meningococcal serogroups by country and region (**Table 1**, **Figure 2**). Due in part to the dynamic nature of *N. meningitidis* serogroup distribution during this time period (Figures S1-S4 in **Online Supplementary Document**), heterogeneity was elevated for many serogroup analyses (Table S4 in **Online Supplementary Document**).

NmW was the predominant circulating serogroup (range: 43.9%-98.2%) throughout most of AFRO, except Algeria, Cameroon, Chad, Guinea and Nigeria. NmA was the most prevalent (range: 76.2%-97.3%) in Cameroon, Chad, and Guinea, while Nigeria was the only country with NmC as the most prevalent (100.0%). The next most prevalent serogroup varied greatly within this region. For example, NmA represented only 0.2% of circulating serogroups in Togo, while NmX represented 22.5% of those within Burkina Faso. Algeria and South Africa were the only countries in AFRO with substantial NmB circulation (59.3% and 29.1%, respectively).

NmB was the most prevalent serogroup in the USA, Canada, Argentina, Colombia, and Uruguay (range: 30.8%-73.0%). Elsewhere in the Americas, either NmC (Brazil, Mexico, and Venezuela; range: 52.8%-88.2%) or NmW (Chile, 51.7%) was the most predominant. The second most prevalent circulating serogroup within AMRO was NmB within most of Latin America (range: 11.8%-37.9%) and NmY within North America (range: 23.6%-25.6%).

Within EURO, NmB predominated in nearly all countries (range: 40.7%-91.7%). In countries where NmB was not the most prominent, NmY was the most prevalent serogroup in Norway and Sweden (37.3% and 42.4%, respectively), NmW in Turkey (47.2% of cases), and NmA in Kyrgyzstan (89.6% of cases).

India was the only country in SEARO included in the analysis, where all reported cases were identified as NmA. Similarly, Sudan was the only country within EMRO included in the analysis. NmA accounted for 90.0% of all IMD cases.

In WPRO, NmB was the primary cause of IMD in both Australia and New Zealand (72.5% and 63.3%, respectively) while, NmC was the prominent circulating serogroup within China (40.3%), and NmY in Japan (61.0%).

We circulated and received a response to participate in our questionnaire-based survey to understand the laboratory capacity for meningococcal surveillance from all WHO regions except AMRO. Response rate was high in EURO, with 67% (4/6) of GISN participating countries responding and 53% (16/30) of ECDC participating countries responding. Similarly, response rate was high in AFRO with 47% (22/47) of countries responding (18 of which are located within the extended meningitis belt). GISN participating countries in WPRO and SEARO also had high response rates at 83% (5/6) and 100% (3/3), respectively. Response rate was lowest among GISN participating countries in EMRO where only 25% (1/4) responded.

Among the participating countries, there were differences in surveillance coverage. Within AFRO, 68% of countries had the entire country under surveillance while none of EMRO and SEARO had countrywide surveillance (**Table 2**). Twenty percent of participants from WPRO had the entire country under surveillance. This number was demonstrably higher within EURO where 89% of the respondents reported country level meningococcal surveillance. Most regions, except AFRO and EURO, primarily focused on surveillance in children younger than five years of age.

**Table 2:** Laboratory capacity for surveillance survey results by country

Surveillance characteristics	Algeria		Burkina Faso		Cameroon		CAR*		Cote d'Ivoire		DRC*		Eritrea		Ethiopia		Ghana		Kenya		Madagascar		Mali		Mauritania		Mozambique		Namibia			Niger		Rwanda		Senegal	South Sudan		Tanzania		Togo		Uganda		Sudan	Azerbaijan		Bulgaria
**Area under surveillance:**																																																
Entire country	✓		✓		✓		✓		✓		✓		✓		✓		✓						✓		✓							✓				✓	✓				✓							✓
Part of Country																			✓		✓						✓		✓					✓					✓				✓		✓	✓		
**Population under surveillance:**																																																
<5 y only																			✓		✓						✓		✓					✓			✓		✓		✓		✓		✓	✓		
<15 y only																																																
<19 y only																																																
<65 only									✓																																							
All ages	✓		✓		✓		✓				✓		✓		✓		✓						✓									✓				✓												✓
Additional High-Risk Groups (if not national surveillance)									✓				✓												✓							✓					✓											
**Surveillance coverage:**																																																
National/population based	✓		✓						✓		✓		✓				✓						nd		✓							✓				✓	✓				nd							✓
Sentinel					✓		✓				✓		✓		✓				✓		✓		nd				✓		✓					✓		✓			✓		nd		✓		✓	✓		
**Type of surveillance:**																																																
Active syndromic	✓								✓		†		✓				nd				✓		✓						nd			✓		✓			†				nd		✓			✓		
Passive syndromic					✓		✓		✓		✓		✓		✓		nd						✓				✓		nd			✓				✓	✓		✓		nd		✓					
Active laboratory confirmed	✓		✓		✓		✓		✓		†		✓				nd				✓		✓						nd			✓		✓			†				nd		✓		✓	✓		
Passive laboratory confirmed					✓		✓		✓		✓		✓		✓		nd		✓				✓		✓		✓		nd			✓				✓	✓		✓		nd		✓					✓
**Length of surveillance (any type):**																																																
<5 y													✓		✓		nd				✓						✓		nd								✓				nd							
5–10 y			✓		✓												nd		✓										nd												nd				✓	✓		
>10 y	✓						✓		✓		✓				‡		nd						✓		✓				nd			✓		✓		✓			✓		nd		✓					✓
**Length of surveillance (laboratory confirmed):**																																																
<5 y													✓		✓		nd				✓						✓		nd								✓				nd							
5–10 y			✓		✓												nd		✓										nd										✓		nd				✓	✓		
>10 y	✓						✓		✓		✓				‡		nd						✓		✓				nd			✓		✓		✓					nd		✓					✓
**Resources for surveillance:**																																																
Government	✓		✓		✓		✓		✓				✓		✓		✓				✓				✓		✓		nd			✓		✓		✓			✓		✓		✓		✓	✓		
Academic	✓						✓																						nd							✓												✓
Private	✓								✓																				nd			✓				✓			✓									
Other (including WHO, Local, research projects, donors, and industry)	✓				✓						✓				✓		✓		✓				✓						nd							✓	✓						✓		✓	✓		
**Purpose of surveillance:**																																																
Disease burden estimates and serogroup distribution	✓		✓		✓		✓				✓		✓		✓		✓		✓		✓		✓		nd		✓		nd			✓		✓		✓	✓		✓		✓		✓		✓	✓		✓
Outbreak detection and prediction	✓		✓		✓		✓		✓		✓		✓		✓		✓		✓		✓		✓		nd		✓		nd			✓				✓	✓				✓		✓			✓		✓
Development of prevention guidelines and vaccination policies			✓		✓				✓				✓		✓		✓		✓		✓		✓		nd		✓		nd			✓				✓	✓		✓		✓		✓			✓		✓
Measure impact of interventions and vaccines			✓		✓				✓		✓				✓		✓		✓		✓		✓		nd		✓		nd			✓		✓		✓			✓		✓		✓		✓			
**Pathogen detection method:**																																																
Multiple tests, including PCR	✓		✓		✓		✓		✓		✓				✓		✓		✓		✓		✓									✓				✓					✓					✓		✓
Multiple tests, excluding PCR													✓												✓		✓		✓					✓			✓		✓				✓		✓			
**Sero/genogrouping frequency:**																																																
All specimens	✓		✓		✓										✓		nd		✓		✓		✓				✓					✓		✓		✓	✓				✓				✓	✓		
Subset of specimens							✓		✓								nd								✓				✓														✓					
Not performed											✓		✓				nd																						✓									✓
**Sero/genogrouping method:**																																																
PCR only			✓																																													
Latex agglutination only																			✓										✓								✓									✓		
Slide agglutionation only																																													✓			
Multiple tests, including PCR	✓				✓		✓		✓						✓		✓						✓				✓					✓				✓					✓							¶
Multiple tests, excluding PCR																									✓									✓									✓					
No laboratory in country													✓								✓																		✓									
Reference laboratory used																	✓				✓						✓										✓						✓		✓	✓		
**Sero/genogrouping global EQA/QC involvement:**																																																
Yes, all laboratories	✓		✓				✓		✓		¶				✓		✓		✓								nd		✓							✓							✓		✓	✓		
Yes, some laboratories					✓																		✓		✓		nd					✓		✓							✓							✓
No involvement																											nd										✓											
No laboratory in country													✓								✓						nd												✓									

**Antimicrobial resistance testing:**																																																
Yes	✓		✓		✓		✓		✓		✓		✓		✓		✓		✓		✓		✓		✓		✓					✓		✓		✓			✓		✓		✓		✓	✓		✓
No																													✓								✓											
Reference laboratory used																																				✓	✓											
**Vaccine use:**																																																
In national immunization schedule			✓				‖		‖								✓								nd				nd			‖													✓			
Selectively for target indications, high-risk groups, recommended, and/or in private sector	✓				✓		✓		✓						✓								✓		nd				nd			✓				✓												✓
In supplementary campaigns			✓		✓		✓		✓		✓		✓		✓								✓		nd				nd			✓				✓	✓						✓					
Not used																			✓		✓				nd		✓		nd					✓					✓		✓					✓		
**National vaccination or prevention guidelines and policies:**																																																
Yes	✓		✓		✓		✓		✓		✓		✓		✓		✓		✓		✓		nd				✓		✓			✓				✓	✓		✓				✓		✓	✓		
No																							nd		✓									✓							✓							✓
**Specific meningococcal disease outbreak guidelines and policies:**																																																
Yes	✓		✓		‖		✓		✓		‖		✓		✓		✓		✓				nd				✓		✓			✓				✓	✓		‖		✓		✓		✓			
No																					✓		nd		✓									✓												✓		✓
**Surveillance characteristics**		**Czech Republic**		**Denmark**		**Estonia**		**Georgia**		**Greece**		**Ireland**		**Latvia**		**Luxembourg**		**Malta**		**Norway**		**Poland**		**Portugal**		**Republic of Armenia**		**Scotland**		**Slovakia**	**Spain**		**Sweden**		**Ukraine**			**Bangladesh**		**Nepal**		**Sri Lanka**	**Cambodia**	**Mongolia**		**Papua New Guinea**	**Philippines**		**Viet Nam**	
**Area under surveillance:**																																																		
Entire country		✓		✓		✓		✓		✓		✓		✓		✓		✓		✓		✓		✓				✓		✓	✓		✓		✓												✓			
Part of country																										✓												✓		✓		✓	✓	✓		✓			✓	
**Population under surveillance:**																																																		
<5 y only																										✓																✓		✓					✓	
<15 y only																																								✓			✓							
<19 y only																																						✓												
<65 only																																																		
All ages		✓		✓		✓		✓		✓		✓		✓		✓		✓		✓		✓		✓				✓		✓	✓		✓		✓											✓	✓			
Additional High-Risk Groups (if not national surveillance)																																			✓															
**Surveillance coverage:**																																																		
National/population based		✓		✓		✓		✓		✓		✓		✓		✓		✓		✓		✓		✓				✓		✓	✓		✓		✓			✓								✓	✓			
Sentinel								✓																		✓									✓			✓		✓		✓	✓	✓		✓	✓		✓	
**Type of surveillance:**																																																		
Active syndromic		✓								✓																		✓							nd			✓		✓		✓		✓		nd				
Passive syndromic		✓		✓								✓		✓		✓						✓									✓		✓		nd			✓					✓	✓		nd	✓			
Active laboratory confirmed		✓		✓						✓								✓						✓				✓		✓					nd			✓						✓		nd			✓	
Passive laboratory confirmed		✓		✓		✓		✓				✓		✓		✓		✓		✓		✓				✓					✓		✓		nd			✓		✓		✓	✓	✓		nd	‡			
**Length of surveillance (any type):**																																																		
<5 y								✓																		nd									nd											nd	✓			
5–10 y								‡																		nd									nd								✓			nd			✓	
>10 y		✓		✓		✓				✓		✓		✓		✓		✓		✓		✓		✓		nd		✓		✓	✓		✓		nd			✓		§		✓		✓		nd				
**Length of surveillance (laboratory confirmed):**																																																		
<5 y								✓																		nd									nd											nd	✓			
5–10 y								‡																		nd									nd								✓			nd			✓	
>10 y		✓		✓		✓				✓		✓		✓		✓		✓		✓		✓		✓		nd		✓		✓	✓		✓		nd			✓		§		✓		✓		nd				
**Resources for surveillance:**																																																		
Government		✓		✓		✓		✓		✓		✓		✓		✓		✓		✓		✓		✓						✓	✓		✓		✓					✓		✓		✓		✓	✓			
Academic																																			✓								✓			✓				
Private										✓																									✓					✓						✓				
Other (including WHO, Local, research projects, donors, and industry)		✓						✓				✓										✓				✓		✓							✓			✓									✓		✓	
**Purpose of surveillance:**																																																		
Disease burden estimates and serogroup distribution		✓		✓		✓		✓		✓		✓		✓		✓		✓		✓		✓		✓		✓		✓		✓	✓		✓		✓			✓		✓		✓	✓	✓		✓	✓		✓	
Outbreak detection and prediction		✓		✓		✓		✓		✓		✓				✓		✓		✓		✓		✓				✓		✓	✓		✓		✓			✓		✓				✓			✓		✓	
Development of prevention guidelines and vaccination policies		✓				✓		✓		✓		✓				✓		✓		✓		✓		✓				✓		✓	✓		✓		✓			✓		✓		✓		✓		✓	✓		✓	
Measure impact of interventions and vaccines		✓				✓		✓		✓		✓				✓				✓				✓				✓		✓	✓							✓		✓		✓	✓	✓			✓		✓	
**Pathogen detection method:**																																																		
Multiple tests, including PCR		✓		✓		✓		✓		✓		✓		✓		✓		✓		✓		✓		✓		✓		✓		✓	✓		✓		✓			✓		✓			✓	✓			✓		✓	
Multiple tests, excluding PCR																																										✓				✓				
**Sero/genogrouping frequency:**																																																		
All specimens		✓		✓		✓				✓		✓		✓		✓		✓		✓		✓		✓		✓		✓			✓		✓		✓			✓		✓		✓		✓					✓	
Subset of specimens								‡																						✓																✓	‡			
Not performed								✓																																			✓				✓			
**Sero/genogrouping method:**																																																	
PCR only																								✓																									✓	
Latex agglutination only																																																		
Slide agglutionation only						✓																																								✓				
Multiple tests, including PCR		✓		✓				‡		✓		✓		✓		✓		✓		✓		✓						✓		✓	✓		✓		✓			✓						✓			‡			
Multiple tests, excluding PCR																																																		
No laboratory in country																										✓														✓		✓	✓			✓				
Reference laboratory used																										✓														✓		✓								
**Sero/genogrouping global EQA/QC involvement:**																																																		
Yes, all laboratories						✓				✓		✓				✓		✓										✓		✓			✓		✓			✓					nd	✓			✓		✓	
Yes, some laboratories		✓		✓				✓												✓		✓		✓							✓												nd							
No involvement														✓																													nd			✓				
No laboratory in country																										✓														✓		✓	nd							
**Antimicrobial resistance testing:**																																																	
Yes		✓		✓		✓		✓		✓		✓		✓		✓		✓		✓		✓		✓				✓		✓	✓		✓					✓		✓		✓	✓	✓			✓		¶	
No																										✓									✓											✓				
Reference laboratory used																																																		
**Vaccine use:**																																																	
In national immunization schedule										✓		✓				✓								✓		✓		✓			✓															nd				
Selectively for target indications, high-risk groups, recommended, and/or in private sector		✓		✓		✓				✓		✓				✓		✓		✓		✓		✓		✓		✓		✓	✓		✓		✓					✓		✓		✓		nd	✓		✓	
In supplementary campaigns												✓										✓						✓			✓															nd				
Not used								✓						✓																								✓					✓			nd				
**National vaccination or prevention guidelines and policies:**																																																	
Yes		✓		✓		✓		✓		✓		✓		✓		✓		✓		✓		✓		✓		✓		✓			✓		✓		✓			✓				✓		✓		✓	✓		✓	
No																														✓										✓			✓							
**Specific meningococcal disease outbreak guidelines and policies:**																																																	
Yes		✓		✓		✓		✓		✓		✓		✓		✓		✓		✓		✓		✓				✓		✓	✓		✓		✓									✓		✓	✓			
No																										✓												✓		✓		✓	✓						✓	

CAR – Central African Republic, DRC – Democratic Republic of the Congo, nd – no data received, EQA – EQA/QC-External quality assurance, QC – quality control, PCR – polymerase chain reaction, WHO – World Health Organization, y – year(s)

*CAR refers to Central African Republic and DRC refers to the Democratic Republic of Congo.

†Only during investigation of outbreaks and/or epidemics.

‡Only at the WHO-supported IB-VPD sentinel site(s).

§Had an interruption in surveillance in the last 5 to 10 years.

‖Select laboratories have the capability but not performed routinely for surveillance. Not yet valid at the national level or in development.

In all respondent countries, surveillance was primarily supported by funding from the government and ‘other’ sources, which included WHO, local support, research projects, donors, and industry grants. Government support ranged from 60% of countries in WPRO to 100% in EMRO and SEARO.

All participating countries utilized surveillance data to primarily better understand the epidemiology of meningococcal disease within their country. A secondary aim of most countries (71%, 35/49) was to measure the impact of interventions and vaccines.

The majority of regions had PCR capabilities for both pathogen detection and serogrouping. Fifty percent of countries within AFRO and SEARO utilized PCR for pathogen detection, while countries within EURO and WPRO utilized PCR more frequently for pathogen detection (100% and 80%, respectively). Use of PCR for genogrouping was high within EURO (75%), while only 30% of countries in AFRO used PCR for genogrouping in-country and 20% utilized a regional reference laboratory for this purpose.

The majority of all regions were regularly performing antimicrobial resistance testing. The specific antibiotics tested for in each region can be found in Table S6 in **Online Supplementary Document**.

Overall, meningococcal vaccine use within the national immunization schedule was low. No countries within SEARO or WPRO included any meningococcal vaccine in a national schedule, and only 13% of AFRO and 35% of EURO did so. Although nationwide use of the vaccine was low, use within high-risk groups or availability within the private sector was higher for EURO, SEARO, and WPRO (80%, 100%, and 75%, respectively). Thirteen percent of countries within AFRO used a meningococcal vaccine in this manner. No use of the vaccine in any form was highest within AFRO (30%), WPRO (25%), and EURO (20%).

## DISCUSSION

Our review demonstrates that there is substantial variability (between countries and WHO regions) in the predominantly circulating meningococcal serogroups. Our survey to determine the laboratory capacity for meningococcal surveillance expands upon previous knowledge and complements the ECDC laboratory capability monitoring system, GISN technical working group meeting reports, and Global Meningococcal Initiative (GMI) roundtable meeting summaries by providing an updated status of meningococcal disease surveillance in comparison to other regions globally [15,16,20-22,30-32]. These results highlight the need for continued efforts in meningococcal surveillance and laboratory capacity to accurately assess the burden of serogroup-specific meningococcal disease and to identify groups at high-risk for meningococcal disease.

Multiple factors within each country can affect the circulating meningococcal serogroups, including vaccine use and outbreaks. Our study showed that serogroup B caused the majority of IMD cases in many countries throughout EURO, WPRO, and AMRO, where the inclusion of either MenC or MenACWY, but not MenB, in the national immunization schedule predominates (data not shown) [33,34]. The experience in the UK highlights how the proportional increase of NmB occurred after introduction of the MenC vaccine. Prior to the introduction of MenC vaccine in the UK in 1999, NmB and NmC caused approximately 50% and 35% of all cases, respectively [35]. Approximately a decade after the introduction of MenC, NmB accounted for 87% of IMD cases in England and Wales, and NmC only 2% [36]. Although overall incidence declined during this period, the high incidence of NmB disease and severe sequelae among infants, led to the introduction of a national MenB vaccine in the UK in 2015 [37]. Similarly, NmA has substantially decreased in AFRO after the introduction of a MenA conjugate vaccine after years of widespread epidemics in the meningitis belt [38,39]. In addition to the influence of national vaccination programs to control previously endemic circulating serogroups such as described above, recent outbreaks have led to the introduction of new national vaccination programs. Examples include increased NmW cases in the UK [40], Chile, and Argentina [15], and the NmC epidemic in Niger (introduction in process, data not shown).

Due to the epidemic potential of meningococcal disease, it is important to establish a systematic surveillance system in each country, or at least in countries prone to large-scale outbreaks, such as in the African meningitis belt. This should be done in order to quickly identify the outbreak; implement effective control measures, such as accessing vaccine stockpiles; and developing preventive policies against the occurrence of outbreaks and control of endemics. For instance, in Chile, increased incidence of NmW and high case fatality rates led to the establishment of mandatory notification surveillance system and targeted vaccination among children [20]. Similarly, Burkina Faso is another striking example of how strategic laboratory capacity development can support the introduction of a vaccine into the national immunization program. From 2007 to 2011, the proportion of isolates received by a reference laboratory for confirmation and serogrouping increased from 11% to approximately 85% [24]. This rapid expansion in capacity for active, cased-based surveillance provided the evidence base to monitor the impact of the MenA vaccine on IMD burden. This was accomplished through assessment, training, mentorship, and technology transfer [38], displaying the benefit of strategic partnerships for capacity development. However, long-term sustainability of the increased capacity is vital in order to see future improvements in surveillance practices. In addition to monitoring vaccine effects, routine surveillance data provide information on the temporal trends of IMD, a dynamic disease. The ability to monitor for outbreaks and determine circulating serogroups and incidence are all dependent on the quality and representativeness surveillance data and laboratory capacity. Our survey results highlight this point as many of the countries that were conducting surveillance in limited populations or sites did not have publically available serogroup data.

Our survey identified the continued need to expand laboratory infrastructure and capacity for identifying meningococcus, serogrouping, and antibiotic susceptibility testing. This is especially necessary to differentiate meningococcal outbreaks from those caused by other organisms, such as *Streptococcus pneumoniae*, as recently seen in Ghana [41]. Ideally the countries with surveillance only in part of the country or passive surveillance, which comprise approximately half of countries participating in this survey, would expand to active, case-based surveillance systems. This would provide a more accurate understanding of serogroup specific disease burden in these countries. Although data from passive surveillance covers many provinces, or sometimes the entire country, these can often be delayed and incomplete. Sentinel surveillance provides some of the necessary details to understand vaccine effects, but does not provide the breadth of coverage as other methods, nor does it allow for understanding of disease incidence. Additionally, the representativeness of sentinel site data are also directly dependent on the size of the catchment area and the proportion of patients with meningococcal disease that reach the sentinel site for treatment. In contrast, national, active case-based surveillance allows for more complete understanding of how interventions, such as vaccine implementation, have affected overall meningococcal incidence and epidemiology. However, we realize this method of surveillance is the most time- and resource-consuming and, therefore, is not currently feasible for many countries, especially those with limited laboratory capacity. In this case, improving the existing sentinel or passive surveillance systems to promote data completeness and timely reporting should be a priority [42].

Strengths of this study include the comprehensive systematic literature review and grey literature search to identify meningococcal serogroup data. Many countries are conducting meningococcal surveillance, but only publish reports on their respective websites. We initially planned to search the Chinese literature databases, but during the hand searching process, national data were identified for China. Therefore, the Chinese databases were not included in this search. An additional strength includes collecting primary meningococcal surveillance data from country-level contacts. Although previous reviews have reported discussions in expert panels and meetings regarding laboratory capacity for meningococcal surveillance [15,16,20-22,32], to our knowledge, this is the first study to provide extensive details about IMD surveillance in several parts of the world.

However, this review is not without its limitations. The quality of the serogroup estimates within this study is highly dependent upon the quality of data available from each country. The sample size that each serogroup distribution estimate is based upon impacts its reliability. Even for countries participating in established surveillance networks, such as SIREVA-II within Latin America, the proportion of isolates from reported cases sent to reference laboratories for characterization is not uniform across all countries, and neither are case definitions or surveillance practices [15,20]. Our decision to only include studies that report serogroup data for at least 15 specimens per year meant exclusion of data from countries that did not meet this eligibility criteria (Table S5 in **Online Supplementary Document**). In addition, dynamic changes in circulating serogroups during the study period due to large epidemics, such as recently reported NmC in Niger and Nigeria [43]; recent natural shifts in predominant serogroups, such as increased NmW in Australia, England, and the Netherlands [44,45]; or vaccine-induced shifts, as in the African meningitis belt [38,39]; would affect the reported serogroup estimates from these countries. Similarly for Benin and India, where only outbreak reports were included in analysis, the serogroup prevalence of the outbreak causing serogroup could be overestimated. It is a known concern that meningococcal data from India heavily relies on data primarily collected during outbreaks, and IMD disease burden could be underestimated in the country [21]. For these reasons, the reported serogroup meta-estimates should be interpreted with caution. We would encourage that the serogroup data presented herein be interpreted together with the reported laboratory capacity data and meningococcal disease incidence data [26]. This can aid policy makers in determining the need for advanced meningococcal surveillance and introduction/scaling up of meningococcal vaccination in their country.

When utilizing surveys for data collection, missing countries and/or missing data may bias the results of the study by not allowing for a complete assessment of surveillance capacity. Additionally, reporting bias could influence the results. We attempted to overcome this limitation by allowing the country-level contacts an opportunity to review interpretation of their data prior to publication. While all countries with WHO and ECDC supported meningococcal surveillance programs were contacted, there are countries outside of these networks that were not asked to participate in this review. Limitations notwithstanding, these results should provide valuable details for countries where this information was previously not publically known. Since this study primarily focused on basic characterization of laboratory capacity, further research is required to determine the advanced capabilities for meningococcal surveillance globally, such as multilocus sequence typing (MLST) or whole genome sequencing (WGS).

## CONCLUSIONS

Our study highlights that the serogroup distribution continues to vary by country and WHO region and reports the laboratory capacity for surveillance in multiple regions. Countries should continue monitoring the circulating meningococcal serogroups and strengthen laboratory capacity as is appropriate in their context. These data can inform disease burden estimates and future vaccination policies or evaluations. Additionally, as molecular testing becomes increasingly more affordable, meningococcal surveillance will continue to play an important role in understanding the emergence and global spread of hypervirulent clonal complexes.

## Additional Material

Online Supplementary Document
