# Supplementary material for: Meningococcal serogroups and surveillance: a systematic review and survey
Source: J Glob Health. 2018 Dec 21;9(1):010409. doi: 10.7189/jogh.09.010409 (PMC6304171; doi:10.7189/jogh.09.010409)
Supplement: Online Supplementary Document [file jogh-09-010409-s001.pdf]

Database Search Strategies

MEDLINE Search Strategy

1. *exp Neisseria meningitidis/*
2. *Neisseria meningitidis.mp.*
3. *Meningitidis.mp.*
4. *Meningitides.mp.*
5. *Neisseria meningitis.mp.*
6. *Meningococc* serogroup*.mp.*
7. *1 or 2 or 3 or 4 or 5 or 6*
8. *Meningitis/*
9. *Meningitis, bacterial/*
10. *Exp meningitis, meningococcal/*
11. *Mengingitis.mp.*
12. *Meningococc*.mp.* → *Captures: Meningococcosis, Meningococc* infection*, Meningococc* disease*, Meningococcal, Meningococcaemia, Meningococcemia, Meningococcus, Invasive Meningococcal Disease*
13. *exp Meningococcal Infections/*
14. *Waterhouse Friderichsen Syndrome.mp.*
15. *Sepsis/*
16. *Sepsis.mp.*
17. *Septic*.mp.* → *Captures: Septic, Septicemia, Septicaemia*
18. *Bacteremia/*
19. *Bacteremia.mp.*
20. *Bacteraemia.mp.*
21. *Neisseriaceae infections/*
22. *Neisseriaceae infection*.mp.*
23. *IMD.mp.*
24. *8 or 9 or 10 or 11 or 12 or 13 or 14 or 15 or 16 or 17 or 18 or 19 or 20 or 21 or 22 or 23*
25. *Serogroup/*
26. *Serogroup*.mp.*
27. *Subtype*.mp.*
28. *Capsular group*.mp.*
29. *Serotyping/*
30. *Serotyp*.mp.*
31. *Epidemiology/*
32. *Epidemiology.mp.*
33. *Meningitis belt.mp.*
34. *Epidemic*.mp.*
35. *Outbreak*.mp.*
36. *25 or 26 or 27 or 28 or 29 or 30 or 31 or 32 or 33 or 34 or 35*
37. *7 and 24 and 36*
38. *limit 37 to (humans and yr="2010-Current")*

1355 results as of 1437 16 October 2017

Embase Search Strategy

1. *exp Neisseria meningitidis/*
2. *Neisseria meningitidis.mp.*
3. *Meningitidis.mp.*
4. *Meningitides.mp.*
5. *Neisseria meningitis.mp.*
6. *Meningococc* serogroup*.mp.*
7. *1 or 2 or 3 or 4 or 5 or 6*
8. *Meningitis/*
9. *Meningitis.mp.*
10. *Bacterial meningitis/*
11. *exp Meningococcosis/*
12. *Meningococc*.mp. → Captures: Meningococcosis, Meningococc* infection*, Meningococc* disease*, Meningococcal, Meningococcaemia, Meningococcemia, Meningococcus, Invasive Meningococcal Disease*
13. *Waterhouse Friderichsen Syndrome.mp.*
14. *Sepsis/*
15. *Sepsis.mp.*
16. *Septic*.mp. → Captures: Septic, Septicemia, Septicaemia*
17. *Bacteremia/*
18. *Bacteremia.mp.*
19. *Bacteraemia.mp.*
20. *Neisseriaceae infection/*
21. *Neisseriaceae infection*.mp.*
22. *IMD.mp.*
23. *8 or 9 or 10 or 11 or 12 or 13 or 14 or 15 or 16 or 17 or 18 or 19 or 20 or 21 or 22*
24. *Serotype/*
25. *Serotyping/*
26. *Serotyp*.mp.*
27. *Serogroup*.mp.*
28. *Subtype*.mp.*
29. *Capsular group*.mp.*
30. *Epidemiology/*
31. *Epidemiology.mp.*
32. *Meningitis belt.mp.*
33. *Epidemic*.mp.*
34. *Outbreak*.mp.*
35. *24 or 25 or 26 or 27 or 28 or 29 or 30 or 31 or 32 or 33 or 34*
36. *7 and 23 and 35*
37. *limit 36 to (humans and yr="2010-Current")*

1734 results as of 1440 16 October 2017

Global Health Database

1. *exp Neisseria meningitidis/*
2. *Neisseria meningitidis.mp.*
3. *Meningitidis.mp.*
4. *Meningitides.mp.*
5. *Neisseria meningitis.mp.*
6. *Meningococcal serogroup$.mp.*
7. *Meningococcus serogroup$.mp.*
8. *1 or 2 or 3 or 4 or 5 or 6 or 7*
9. *Meningitis/*
10. *Bacterial meningitis/*
11. *Mengingitis.mp.*
12. *Meningococcosis.mp.*
13. *Meningococcal.mp.*
14. *Meningococcus.mp.*
15. *Meningococci.mp.*
16. *Meningococc?emia.mp.*
17. *Waterhouse Friderichsen Syndrome.mp.*
18. *exp Sepsis/*
19. *Sepsis.mp.*
20. *Septic$.mp.* → *Captures: Septic, Septicemia, Septicaemia*
21. *Bacteraemia/*
22. *Bacter?emia.mp.*
23. *Neisseriaceae infection$.mp.* → *No results found*
24. *IMD.mp.*
25. *9 or 10 or 11 or 12 or 13 or 14 or 15 or 16 or 17 or 18 or 19 or 20 or 21 or 22 or 23 or 24*
26. *Serogroup$.mp.*
27. *Subtype$.mp.*
28. *Capsular group$.mp.*
29. *Serotypes/*
30. *Serotyp$.mp.*
31. *Epidemiology/*
32. *Epidemiology.mp.*
33. *Meningitis belt.mp.*
34. *Epidemics/*
35. *Epidemic$.mp.*
36. *Outbreaks/*
37. *Outbreak$.mp.*
38. *26 or 27 or 28 or 29 or 30 or 31 or 32 or 33 or 34 or 35 or 36 or 37*
39. *8 and 25 and 38*
40. *limit 39 to (humans and yr="2010-Current")*

1712 Results as of 1447 16 October 2017

Web of Science Search Strategy (free text searches)

((Neisseria meningitidis) or (meningitidis) or (Neisseria meningitis) or (Meningitides) or (Meningococc serogroup*)) AND ((Mengingitis) or (Meningococc*) or (Waterhouse Friderichsen Syndrome) or (Sepsis) or (Septic*) or (Bacter$emia) or (Neisseriaceae infection*) or (IMD)) AND ((Serogroup*) or (Subtype*) or (Capsular group*) or (Serotyp*) or (Epidemiology) or (Meningitis belt) or (Epidemic*) or (Outbreak*))*

Date Limiters: 2010 to 2017

1593 Results as of 1501 16 October 2017

Current Contents Connect Search

((Neisseria meningitidis) or (meningitidis) or (Neisseria meningitis) or (Meningitides) or (Meningococc serogroup*)) AND ((Mengingitis) or (Meningococc*) or (Waterhouse Friderichsen Syndrome) or (Sepsis) or (Septic*) or (Bacter$emia) or (Neisseriaceae infection*) or (IMD)) AND ((Serogroup*) or (Subtype*) or (Capsular group*) or (Serotyp*) or (Epidemiology) or (Meningitis belt) or (Epidemic*) or (Outbreak*))*

Date Limiters: 2010 to 2017

1116 Results as of 1508 16 October 2017

Global Health Library

Includes: AIM (AFRO), LILACS (AMRO/PAHO), IMEMR (EMRO), IMSEAR (SEARO), WPRIM (WPRO), WHOLIS (KMS)

((Neisseria meningitidis) or (meningitidis) or (Neisseria meningitis) or (Meningitides) or (Meningococc serogroup*)) AND ((Mengingitis) or (Meningococc*) or (Waterhouse Friderichsen Syndrome) or (Sepsis) or (Septic*) or (Bacter$emia) or (Neisseriaceae infection*) or (IMD)) AND ((Serogroup*) or (Subtype*) or (Capsular group*) or (Serotyp*) or (Epidemiology) or (Meningitis belt) or (Epidemic*) or (Outbreak*))*

Filters: 2010, 2011, 2012, 2013, 2014, 2015, 2016, 2017

127 Results as of 1514 16 October 2017

Table S1. African Meningitis Belt Comparisons.

Country	Study	Year	Reason Selected Study was Chosen
**Burkina Faso**	**Ky-Ba A, Sanou M, Tranchot JD, Christiasen PA, Ouedraogo AS, Tamboura Met al. (1)**	**Jan 2010 to Dec 2010**	Ky-Ba et al was the most representative study with the largest sample size.
	Delrieu I, Yaro S, Tamekloé TAS, Njanpop-Lafourcade BM, Tall H, Jaillard Pet al. (2)	Jan 2010 to Dec 2010 (outbreak)	
	Delrieu I, Yaro S (2)	Jan 2010 to Dec 2010 (surveillance)	
	Novak RT, Kambou JL, Diomandé FVK, Tarbangdo TF, Ouédraogo-Traoré R, Sangaré Let al. (3)	Jan 2010 to Dec 2010	
	Intercountry Support Team—West Africa (4)	Jan 2010 to Dec 2010	
	**Ky-Ba A, Sanou M (1)**	**Jan 2011 to Dec 2011**	Ky-Ba et al was the most representative study with the largest sample size that was not represented in a bar chart (Novak). Author contact was attempted to acquire the raw data from Novak with no response.
	Novak RT, Kambou JL (3)	Jan 2011 to Dec 2011	
	Intercountry Support Team—West Africa (5)	Jan 2011 to Dec 2011	
	**MacNeil JR, Medah I, Koussoube D, Novak RT, Cohn AC, Diomande FVKet al. (6)**	**Jan 2012 to Dec 2012**	MacNeil et al was the most representative study with the largest sample size.
	Cibrelus L, Medah I, Koussoubé D, Yélbeogo D, Fernandez K, Lingani Cet al. (7)	Jan 2012 to Apr 2012	
	Ky-Ba A, Sanou M (1)	Jan 2012 to Dec 2012	
	Savadogo M, Kyélem N, Yelbeogo D, Koussoubé D, Tarbagdo F and Ouédraogo A (8)	Jan 2012 to Apr 2012	
	**Ky-Ba A, Sanou M (1)**	**Jan 2013 to Dec 2013**	Ky-Ba et al was the most representative study with the largest sample size.
	Intercountry Support Team—West Africa (9)	Jan 2013 to Dec 2013	
	**Ky-Ba A, Sanou M (1)**	**Jan 2014 to Dec 2014**	Ky-Ba et al was the most representative study with the largest sample size.
	Intercountry Support Team—West Africa (10)	Jan 2014 to Dec 2014	
**Cameroon**	**Massenet D, Birguel J, Azowé F, Ebong C, Gake B, Lombart JPet al. (11)**	**Jan 2010 to Dec 2010**	Although representing a region of Cameroon, Massenet et al (2013) had a larger sample size than WHO (2010) and Massenet et al (2011). This study still represented a large representation of Cameroon.
	Massenet D, Vohod D, Hamadicko H and Caugant DA (12)	Jan 2010 to Dec 2010	
	Intercountry Support Team—West Africa (4)	Jan 2010 to Dec 2010	

**Chad**	**Intercountry Support Team—West Africa (4)**	**Jan 2010 to Dec 2010**	WHO had a larger sample size than Gamougam.
	Gamougam K, Daugla DM, Toralta J, Ngadoua C, Fermon F, Page ALet al. (13)	Jan 2010 to Jun 2010	
	**Gamougam K, Daugla DM (13)**	**Jan 2011 to Jun 2011**	Gamougam had a larger sample size than WHO.
	Intercountry Support Team—West Africa (5)	Jan 2011 to Sep 2011	
	**Daugla DM, Gami JP, Gamougam K, Naibei N, Mbainadji L, Narbé Met al. (14)**	**Jan 2011 to Jun 2012**	Daugla et al and Gamougam et al reported the same data. One had to be selected over the other, but either would have been acceptable. They both had a greater samples size than WHO.
	Gamougam K, Daugla DM (13)	Jan 2011 to Jun 2012	
	Intercountry Support Team—West Africa (15)	Jan 2012 to Nov 2012	
**The Gambia**	**Intercountry Support Team—West Africa (15)**	**Jan 2012 to Dec 2012**	Although the Hossain articles had a much larger sample size, the WHO surveillance report is more representative than the outbreak reports.
	Hossain MJ, Roca A, Mackenzie GA, Jasseh M, Hossain I, Muhammad Set al. (16)	Feb 2012 to Jun 2012	
	Hossain MJ, Roca A, Mackenzie GA, Jasseh M, Hossain MI, Muhammad Set al. (17)	Feb 2012 to Jun 2012	
**Ghana**	**Intercountry Support Team—West Africa (18)**	**Jan 2015 to Dec 2015**	The WHO reports covered a greater time period.
	**Intercountry Support Team—West Africa (19)**	**Jan 2016 to Dec 2016**	
	Kwambana-Adams BA, Asiedu-Bekoe F, Sarkodie B, Afreh OK, Kuma GK, Owusu-Okyere Get al. (20)	Dec 2015 to Apr 2016	
**Mali**	**Intercountry Support Team—West Africa (19)**	**Jan 2016 to Dec 2016**	WHO covered a greater time period.
	MenAfriNet (21)	Jan 2016 to Mar 2016	
**Niger**	**Collard JM, Issaka B, Zaneidou M, Hugonnet S, Nicolas P, Taha MKet al. (22)**	**Jan 2010 to Dec 2010**	Collard et al had the larger sample size.
	Intercountry Support Team—West Africa (4)	Jan 2010 to Dec 2010	
	**Collard JM, Issaka B (22)**	**Jan 2010 to Dec 2011**	Collard et al had the larger sample size.

	Intercountry Support Team—West Africa (5)	Jan 2011 to Nov 2011	
**Senegal**	**Intercountry Support Team—West Africa (15)**	Jan 2012 to Dec 2012	Although Ndow et al had a much larger sample size, the WHO surveillance report is more representative than the conference abstract as it reports on more than one serogroup. Although Ba et al had a larger sample size than the WHO report, the number of cases for each serogroup were unclear as the reported cases in the text did not match the graphs.
	Ndow G, Manga NM, Ba IO, Ka D, Cisse-Diallo V, Diop SAet al. (23)	2012	
	Ba ID, Deme-Ly I, Thiongane A, Diop A, Sonko A, Keita LMet al. (24)	Jan 2012 to Dec 2012	
**Togo**	**Intercountry Support Team—West Africa (19)**	**Jan 2016 to Dec 2016**	WHO had a larger sample size and represented a longer time period.
	MenAfriNet (21)	Jan 2016 to Mar 2016	
Studies listed in **bold** were selected for inclusion in the meta-analysis.			

Table S2. Laboratory Capacity Questionnaire.

**Name of country**			
**Pathogens routinely tested for as part of bacterial meningitis surveillance**			
***Neisseria meningitidis* (Nm)**			
***Streptococcus pneumoniae* (Sp)**			
***Haemophilus influenzae* (Hi)**			
**Area under surveillance**	**Nm**	**Sp**	**Hi**
Entire country			
Part of country (use space below to name all cities/districts/provinces included)			
**Population under surveillance**	**Nm**	**Sp**	**Hi**
<5 yrs			
5-19 yrs			
20-64 yrs			
>65 yrs			
High risk groups – military/police, college/university students (Specify)			
**Are you part of a surveillance network? If yes, please name**			
**Type of surveillance**	**Nm**	**Sp**	**Hi**
Type of surveillance (Indicate whether sentinel/ population based/ national)			
Active surveillance (syndromic) (also indicate date of start)			
Passive surveillance (syndromic) (also indicate date of start)			
Active surveillance (Lab confirmed) (also indicate date of start)			
Passive surveillance (Lab confirmed) (also indicate date of start)			
**Pathogen detection (check all that apply)**	**Nm**	**Sp**	**Hi**
PCR			
Culture			
Latex agglutination			
Antigen detection (Binax)			
Other (specify)			
**Number and names of labs in country that are capable of pathogen detection (use space below for names of labs)**			
**Number and names of labs involved in pathogen detection that are part of global EQA/QC process (use below for names of labs)**			

Is serogrouping/serotyping routinely performed?	Nm	Sp	Hi
On all specimens			
On a subset (specify)			
Not performed			

Number and names of labs in country that are capable of serogrouping (use space below for names of labs)

If no labs in country, where do you send for serogrouping?

Number and names of labs involved in serogrouping that are part of global EQA/QC process (use space below for names of labs)

Serogrouping/ Serotyping procedures (check all that apply)	Nm	Sp	Hi
Quellung			
Slide agglutination			
Latex agglutination			
PCR			
Other (specify)			

Are serogrouping and serotyping performed together? If yes, skip the next section.	
Yes	
No	

Serotyping

Number and names of labs in country that are capable of serotyping (use space below for names of labs)

If no labs in country, where do you send for serotyping?

Number and names of labs involved in serotyping that are part of global EQA/QC process (use space below for names of labs)

Antimicrobial Resistance (AMR)

Number and names of labs in country capable of testing for AMR in *N. meningitidis* (use space below for names of labs)

Number and names of labs in country capable of testing for AMR in *S. pneumoniae* (use space below for names of labs)

If no labs in country, where do you send for AMR?

Which antibiotics are tested for AMR in *N. meningitidis*?

Which antibiotics are tested for AMR in *S. pneumoniae*?

AMR detection method (check all that apply)	Nm	Sp
Broth dilution MIC		
Agar dilution MIC		
Disk diffusion		
Antimicrobial gradient method/ Etest		
Automated instruments		
Other (specify)		
AMR not performed		

Type of resources (check all that apply)	Nm	Sp	Hi
Government lead			
Academic lead			
Private lead			
Other (specify)			

Purpose of systems (check all that apply)	Nm	Sp	Hi
Disease burden estimates/presence of disease/serogroup and serotype distribution			
Outbreak detection/prediction			
Develop prevention guidelines/policies for vaccination			
Measure for the impact of interventions – vaccination			

Is a meningococcal vaccine being used?	
Routinely	
Selectively (for target indications)	
In supplementary immunization campaigns	
Specify which vaccines are in use:	

Is a pneumococcal vaccine being used?			
Routinely			
Selectively (for target indications)			
In supplementary immunization campaigns			
Specify which vaccines are in use:			
**Is a Hib vaccine being used?**			
Routinely			
Selectively (for target indications)			
In supplementary immunization campaigns			
Specify which vaccines are in use:			
**What is the scale of vaccination use?**	**Nm**	**Sp**	**Hi**
National EPI program (indicate type of vaccine and date started)			
Private sector (indicate type of vaccine and date started)			
Estimated vaccine uptake			
**Are there any prevention guidelines/policies for vaccination? If yes, please provide details.**			
**Do you have national guidelines / recommendations for control of meningococcal meningitis outbreaks? If yes, please provide details.**			
**What are the specimen storage procedures at surveillance sites?**			
**What are the specimen shipping procedures?**			

Table S3. Summary of Serogroup Data by Study. If supplementary data were received from the author, the study is marked with an (*). The serogroups with assumed zeroes are marked with an (†).

Study	Country/Countries	Year(s) Included	Age Group	Sample Size	Contribution To Analysis
**African Region (AFRO)**					
**Réseau Algérien de Surveillance de la Résistance des Bactéries aux Antibiotiques (AARN) (25)**	Algeria	2010	All Ages	25	NmA, NmB, NmC, NmW, NmY, Other
**Réseau Algérien de Surveillance de la Résistance des Bactéries aux Antibiotiques (AARN) (26)**	Algeria	2011	All Ages	24	NmA, NmB, NmC, NmW, NmY, Other
**Réseau Algérien de Surveillance de la Résistance des Bactéries aux Antibiotiques (AARN) (27)**	Algeria	2015	All Ages	22	NmA, NmB, NmC, NmW, NmY
**Njanpop-Lafourcade BM, Hugonnet S, Djogbe H, Kodjo A, N'Douba AK, Taha MKet al. (28)**	Benin<br>(Northern Benin)	Jan–Jun 2012	All Ages	71	NmW, NmX
**Ky-Ba A, Sanou M (1)**	Burkina Faso	2010, 2011, 2013, 2014	All Ages	2010: 229<br>2011: 273<br>2013: 259<br>2014: 215	NmA, NmW, NmX
**MacNeil JR, Medah I (6)**	Burkina Faso	2012	All Ages	1663	NmB, NmC, NmW, NmX
**Massenet D, Birguel J (11)**	Cameroon<br>(Norther Cameroon: Far North, North, and Adamawa)	2010	All Ages	41	NmA, NmB†, NmC†, NmW, NmX†, NmY†
**Gamougam K, Daugla DM (13)**	Chad	Jan–Jun 2011	All Ages	159	NmA, NmW, NmX
**Daugla DM, Gami JP (14)**	Chad	Jan–Jun 2012	All Ages	65	NmA, NmW, NmX
**Mihret W, Lema T, Merid Y, Kassu A, Abebe W, Moges Bet al. (29)**	Ethiopia (Gondar, Addis Ababa, and Hawassa)	Feb 2012–Jun 2013	All Ages	27	NmA, NmB, NmC, NmW, NmX, NmY, Other
**Collard JM, Issaka B (22)**	Niger	2010, 2011	All Ages	2010: 921<br>2011: 410	NmA, NmB†, NmC†, NmW, NmX†, NmY, Other
**National Institute for Communicable Diseases (NICD) (30)**	South Africa	2010	All Ages	334	NmA, NmB, NmC, NmW, NmX, NmY
**National Institute for Communicable Diseases (NICD) (31)**	South Africa	2011	All Ages	275	NmA, NmB, NmC, NmW, NmX, NmY
**National Institute for Communicable Diseases (NICD) (32)**	South Africa	2012	All Ages	176	NmA, NmB, NmC, NmW, NmX†, NmY, Other
**National Institute for Communicable Diseases (NICD) (33)**	South Africa	2013	All Ages	190	NmA, NmB, NmC, NmW, NmX†, NmY, Other
**National Institute for Communicable Diseases (NICD) (34)**	South Africa	2014	All Ages	156	NmA, NmB, NmC, NmW, NmX, NmY, Other
**National Institute for Communicable Diseases (NICD) (35)**	South Africa	2015	All Ages	127	NmA, NmB, NmC, NmW, NmX, NmY, Other
**Intercountry Support Team—West Africa (4)**	Chad, Ghana, Mali, Nigeria	2010	All Ages	Chad: 62<br>Ghana: 28<br>Mali: 36<br>Nigeria: 101	Chad, Ghana, Mali: NmA, NmB, NmC, NmW, NmX, NmY<br>Nigeria: NmA, NmW
**Intercountry Support Team—West Africa (5)**	Cameroon, Mali	Cameroon: Jan–Jul 2011<br>Mali: Jan–Nov 2011	All Ages	Cameroon: 92<br>Mali: 29	NmA, NmB, NmC, NmW, NmX, NmY

**Intercountry Support Team—West Africa (15)**	Cameroon, Cote d'Ivoire, The Gambia, Ghana, Mali, Niger, Senegal	Cameroon: Jan–May 2012<br>Cote d'Ivoire: 2012<br>Gambia: 2012<br>Ghana: 2012<br>Mali: 2012<br>Niger: 2012<br>Senegal: 2012	All Ages	Cameroon: 19<br>Cote d'Ivoire: 90<br>Gambia: 28<br>Ghana: 32<br>Mali: 94<br>Niger: 22<br>Senegal: 22	NmA, NmB, NmC, NmW, NmX, NmY
**Intercountry Support Team—West Africa (9)**	Ghana, Guinea	Ghana: 2013<br>Guinea: 2013	All Ages	Ghana: 18<br>Guinea: 15	NmA, NmB, NmC, NmW, NmX, NmY
**Intercountry Support Team—West Africa (10)**	Ghana, Niger, Nigeria	2014	All Ages	Ghana: 45<br>Niger: 24<br>Nigeria: 38	NmA, NmB, NmC, NmW, NmX, NmY
**Intercountry Support Team—West Africa (18)**	Burkina Faso, Ethiopia, Ghana, Guinea, Mali, Niger, Nigeria, Togo	Burkina Faso: 2015<br>Ethiopia: Jan–Nov 2015<br>Ghana: 2015<br>Guinea: Jan–Oct 2015<br>Mali: 2015<br>Niger: 2015<br>Nigeria: Jan–Oct 2015<br>Togo: 2015	All Ages	Burkina Faso: 258<br>Ethiopia: 16<br>Ghana: 34<br>Guinea: 74<br>Mali: 23<br>Niger: 1390<br>Nigeria: 20<br>Togo: 36	NmA, NmB, NmC, NmW, NmX, NmY
**Intercountry Support Team—West Africa (19)**	Burkina Faso, Central African Republic, Ghana, Mali, Niger, Togo	2016	All Ages	Burkina Faso: 176<br>Central African Republic: 54<br>Ghana: 201<br>Mali: 44<br>Niger: 352<br>Togo: 307	NmA, NmB, NmC, NmW, NmX, NmY
**Region of the Americas (AMRO)**					
**Regueira M and Corso A (36)**	Argentina	2015	All Ages	93	NmA†, NmB, NmC, NmW, NmX†, NmY, Other
**Regueira M, Corso A, Efron A and Gagetti P (37)**	Argentina	2016	All Ages	80	NmA, NmB, NmC, NmW, NmX, NmY, Other
**Silva de Lemos A, Outeiro Gorla M, Cobo Zanella R, Grassi Almeida S, Bokermann S and De Cunto Brandileone M (38)**	Brazil	2015	All Ages	218	NmA, NmB, NmC, NmW, NmX, NmY, Other
**Li YA, Tsang R, Desai S and Deehan H (39)***	Canada	2010, 2011	All Ages	2010: 143<br>2011: 163	NmA, NmB, NmC, NmW, NmX, NmY, Other
**Tsang RS, Law DK, Deng S and Hoang L (40)**	Canada	2013–2015	All Ages	277	NmA†, NmB, NmC, NmW, NmX†, NmY, Other
**Instituto de Salud Pública de Chile (ISP) (41)**	Chile	2013	All Ages	136	NmA†, NmB, NmC, NmW, NmX†, NmY, Other
**Instituto de Salud Pública de Chile (ISP) (42)**	Chile	2014	All Ages	140	NmA†, NmB, NmC, NmW, NmX†, NmY, Other
**Instituto de Salud Pública de Chile (ISP) (43)**	Chile	2015	All Ages	120	NmA†, NmB, NmC, NmW, NmX†, NmY, Other
**Instituto de Salud Pública de Chile (ISP) (44)**	Chile	2016	All Ages	106	NmA†, NmB, NmC, NmW, NmX†, NmY, Other
**Centers for Disease Control and Prevention (CDC) (45)***	United States	2010	All Ages	56	NmA, NmB, NmC, NmW, NmY, Other
**Centers for Disease Control and Prevention (CDC) (46)***	United States	2011	All Ages	75	NmA, NmB, NmC, NmW, NmY, Other
**Centers for Disease Control and Prevention (CDC) (47)***	United States	2012	All Ages	57	NmA, NmB, NmC, NmW, NmY, Other
**Centers for Disease Control and Prevention (CDC) (48)***	United States	2013	All Ages	53	NmA, NmB, NmC, NmW, NmY, Other

**Centers for Disease Control and Prevention (CDC) (49)***	United States	2014	All Ages	53	NmA, NmB, NmC, NmW, NmY, Other
**Centers for Disease Control and Prevention (CDC) (50)***	United States	2015	All Ages	46	NmA[†], NmB, NmC, NmW, NmY, Other
**Pan American Health Organization (PAHO) (51)**	Argentina, Brazil, Chile, Colombia, Mexico, Uruguay, Venezuela	2010	All Ages	Argentina: 134<br>Brazil: 645<br>Chile: 56<br>Colombia: 17<br>Mexico: 17<br>Uruguay: 20<br>Venezuela: 18	NmA, NmB, NmC, NmW, NmX, NmY, Other
**Pan American Health Organization (PAHO) (52)**	Argentina, Brazil, Chile, Colombia, Uruguay, Venezuela	2011	All Ages	Argentina: 152<br>Brazil: 582<br>Chile: 62<br>Colombia: 25<br>Uruguay: 27<br>Venezuela: 26	Argentina: NmA[†], NmB, NmC, NmW, NmX[†], NmY, Other<br>Brazil: NmA[†], NmB, NmC, NmW, NmX[†], NmY, Other<br>Chile: NmA[†], NmB, NmC, NmW, NmX[†], NmY, Other[†]<br>Colombia: NmA[†], NmB, NmC, NmW, NmX[†], NmY, Other[†]<br>Uruguay: NmA[†], NmB, NmC, NmW, NmX[†], NmY, Other[†]<br>Venezuela: NmA[†], NmB, NmC, NmW[†], NmX[†], NmY, Other
**Pan American Health Organization (PAHO) (53)**	Argentina, Brazil, Chile, Colombia, Uruguay, Venezuela	2012	All Ages	Argentina: 173<br>Brazil: 513<br>Chile: 100<br>Colombia: 30<br>Uruguay: 22<br>Venezuela: 28	Argentina: NmA[†], NmB, NmC, NmW, NmX[†], NmY, Other<br>Brazil: NmA[†], NmB, NmC, NmW, NmX[†], NmY, Other[†]<br>Chile: NmA[†], NmB, NmC, NmW, NmX[†], NmY, Other[†]<br>Colombia: NmA[†], NmB, NmC, NmW[†], NmX[†], NmY, Other[†]<br>Uruguay: NmA[†], NmB, NmC, NmW[†], NmX[†], NmY[†], Other<br>Venezuela: NmA[†], NmB, NmC, NmW[†], NmX[†], NmY, Other[†]
**Pan American Health Organization (PAHO) (54)**	Argentina, Brazil, Colombia, Venezuela	2013	All Ages	Argentina: 155<br>Brazil: 384<br>Colombia: 34<br>Venezuela: 24	Argentina: NmA[†], NmB, NmC, NmW, NmX, NmY, Other[†]<br>Brazil: NmA[†], NmB, NmC, NmW, NmX, NmY, Other<br>Colombia: NmA[†], NmB, NmC, NmW[†], NmX[†], NmY, Other<br>Venezuela: NmA[†], NmB, NmC, NmW, NmX[†], NmY, Other
**Pan American Health Organization (PAHO) (55)**	Argentina, Brazil, Colombia, Venezuela	2014	All Ages	Argentina: 149<br>Brazil: 308<br>Colombia: 41<br>Venezuela: 16	Argentina: NmA[†], NmB, NmC, NmW, NmX[†], NmY, Other[†]<br>Brazil: NmA[†], NmB, NmC, NmW, NmX[†], NmY, Other<br>Colombia: NmA[†], NmB, NmC, NmW[†], NmX[†], NmY, Other[†]<br>Venezuela: NmA[†], NmB, NmC, NmW[†], NmX[†], NmY[†], Other[†]
**Eastern Mediterranean Region (EMRO)**					
**Razki A, Zerouali K, Belabbas H, Bouayad A, Elmdaghri N, Deghmane AEet al. (56)**	Morocco	2011–2016	All Ages	143	NmB, NmC, NmW, NmY
**Intercountry Support Team—West Africa (15)**	Sudan (located within the African Meningitis Belt)	Jan–Jun 2012	All Ages	20	NmA, NmB, NmC, NmW, NmX, NmY
**European Region (EURO)**					
**National Reference for Meningococcal Disease (57)**	Austria	2010	All Ages	80	NmA[†], NmB, NmC, NmW, NmX[†], NmY, Other[†]
**National Reference for Meningococcal Disease (58)**	Austria	2011	All Ages	60	NmA[†], NmB, NmC, NmW, NmX[†], NmY, Other
**National Reference for Meningococcal Disease (59)**	Austria	2012	All Ages	50	NmA[†], NmB, NmC, NmW, NmX[†], NmY, Other
**National Reference for Meningococcal Disease (60)**	Austria	2013	All Ages	55	NmA[†], NmB, NmC, NmW, NmX[†], NmY, Other[†]
**National Reference for Meningococcal Disease (61)**	Austria	2014	All Ages	35	NmA, NmB, NmC, NmW, NmX, NmY, Other
**National Reference for Meningococcal Disease (62)**	Austria	2015	All Ages	23	NmA, NmB, NmC, NmW, NmX, NmY, Other
**National Reference for Meningococcal Disease (63)**	Austria	2016	All Ages	31	NmA, NmB, NmC, NmW, NmX, NmY, Other
**Bertrand S, Carion F and Stragier P (64)**	Belgium	2010	All Ages	96	NmA, NmB, NmC, NmW, NmX, NmY, Other
**Bertrand S, Mattheus W, Vanhoof R and Carion F (65)**	Belgium	2011	All Ages	112	NmA, NmB, NmC, NmW, NmX, NmY, Other

**Bertrand S, Mattheus W and Vanhoof R (66)**	Belgium	2012	All Ages	123	NmA, NmB, NmC, NmW, NmX, NmY, Other
**Bertrand S, Mattheus W and Vanhoof R (67)**	Belgium	2013	All Ages	134	NmA, NmB, NmC, NmW, NmX, NmY, Other
**Bertrand S, Mattheus W and Vanhoof R (68)**	Belgium	2014	All Ages	87	NmA, NmB, NmC, NmW, NmX, NmY, Other
**Bertrand S, Mattheus W, Vanhoof R and Ceyssens P (69)**	Belgium	2015	All Ages	98	NmA, NmB, NmC, NmW, NmX, NmY, Other
**Čeljuska-Tošev E, Bukovski-Simonoski S, Gužvinec M, Kovačević G and Knezović I (70)**	Croatia (Zagreb)	2010	All Ages	24	NmB, NmC, NmW, NmY
**Bröker M, Bukovski S, Culic D, Jacobsson S, Koliou M, Kuusi Met al. (71)**	Croatia	2012	All Ages	20	NmB
**Bröker M, Emonet S, Fazio C, Jacobsson S, Koliou M, Kuusi Met al. (72)**	Croatia	2013	All Ages	26	NmB
**Křížová P, Musílek M, Vacková Z and Kozáková J (73)**	Czech Republic	2010	All Ages	51	NmA, NmB, NmC, NmW, NmX[†], NmY
**Křížová P, Musílek M, Vacková Z and Kozáková J (74)**	Czech Republic	2011	All Ages	54	NmA[†], NmB, NmC, NmW, NmX, NmY
**Křížová P, Musílek M, Vacková Z and Kozáková J (75)**	Czech Republic	2012	All Ages	58	NmA, NmB, NmC, NmW, NmX[†], NmY
**Křížová P, Musílek M, Vacková Z and Kozáková J (76)**	Czech Republic	2013	All Ages	51	NmA[†], NmB, NmC, NmW, NmX, NmY
**Křížová P, Musílek M, Vacková Z, Bečvářová Z, Kozáková J and Šebestová H (77)**	Czech Republic	2014	All Ages	37	NmA[†], NmB, NmC, NmW, NmX, NmY
**Křížová P, Musílek M, Vacková Z, Bečvářová Z, Kozáková J and Šebestová H (78)**	Czech Republic	2015	All Ages	46	NmA[†], NmB, NmC, NmW, NmX[†], NmY, Other
**Křížová P, Musílek M, Vacková Z, Jandová Z, Kozáková J and Šebestová H (79)**	Czech Republic	2016	All Ages	39	NmA[†], NmB, NmC, NmW, NmX[†], NmY
**Rasmussen JN, Valentiner-Branth P, Hoffmann S and Mygh A (80)**	Denmark	2010	All Ages	65	NmA, NmB, NmC, NmW, NmX, NmY, Other
**Bjerre C, Valentiner-Branth P, Dalby T and Hoffmann S (81)**	Denmark	2011	All Ages	84	NmA, NmB, NmC, NmW, NmX, NmY, Other[†]
**Suppli CH, Valentiner-Branth P and Hoffmann S (82)**	Denmark	2012	All Ages	55	NmA, NmB, NmC, NmW, NmX, NmY, Other[†]
**Suppli CH, Valentiner-Branth P and Hoffmann S (83)**	Denmark	2013	All Ages	44	NmA, NmB, NmC, NmW, NmX, NmY, Other[†]
**Voss S, Suppli CH, Valentiner-Branth P and Hoffmann S (84)**	Denmark	2014	All Ages	40	NmA, NmB, NmC, NmW, NmX, NmY, Other[†]
**Espenhain L, Suppli CH, Valentiner-Branth P, Fuursted K and Hoffmann S (85)**	Denmark	2015	All Ages	25	NmA, NmB, NmC, NmW, NmX, NmY, Other
**Valentiner-Branth P, Andersen P, Christiansen A, Krause T, Ertner G, Nørgaard Jet al. (86)**	Denmark	2016	All Ages	28	NmA[†], NmB, NmC[†], NmW, NmX, NmY, Other
**National Institute for Health and Welfare (THL) (87)**	Finland	2010	All Ages	32	NmA, NmB, NmC, NmW, NmY
**National Institute for Health and Welfare (THL) (88)**	Finland	2011	All Ages	33	NmA, NmB, NmC, NmW, NmY
**National Institute for Health and Welfare (THL) (89)**	Finland	2012	All Ages	29	NmA, NmB, NmC, NmW, NmY
**National Institute for Health and Welfare (THL) (90)**	Finland	2013	All Ages	20	NmA, NmB, NmC, NmW, NmY

**National Institute for Health and Welfare (THL) (91)**	Finland	2014	All Ages	18	NmA, NmB, NmC, NmW, NmY
**National Institute for Health and Welfare (THL) (92)**	Finland	2015	All Ages	20	NmA, NmB, NmC, NmW, NmY
**Toropainen M, Vainio A and Kuusi M (93)**	Finland	2016	All Ages	18	NmA†, NmB, NmC, NmW, NmY
**Châtelet IPd, Taha MK, Lepoutre A, Maine C, Deghmane AE and Lévy-Bruhl D (94)**	France	2010	All Ages	483	NmA, NmB, NmC, NmW, NmX, NmY, Other
**Châtelet IPd, Taha MK, Lepoutre A, Maine C, Deghmane AE and Lévy-Bruhl D (95)**	France	2011	All Ages	530	NmA, NmB, NmC, NmW, NmX, NmY, Other
**Barret AS, Deghmane AE, Lepoutre A, Fonteneau L, Maine C, Taha MKet al. (96)***	France	2012	All Ages	539	NmA†, NmB, NmC, NmW, NmX†, NmY, Other
**Santé Publique France (97)***	France	2013	All Ages	561	NmA†, NmB, NmC, NmW, NmX, NmY, Other
**Santé Publique France (98)**	France	2014	All Ages	412	NmA†, NmB, NmC, NmW, NmX, NmY, Other†
**Santé Publique France (99)**	France	2015	All Ages	453	NmA†, NmB, NmC, NmW, NmX, NmY, Other
**Santé Publique France (100)**	France	2016	All Ages	506	NmA†, NmB, NmC, NmW, NmX, NmY, Other
**Robert Koch Insitut (RKI) (101)**	Germany	2010	All Ages	344	NmA, NmB, NmC, NmW, NmX†, NmY, Other
**Robert Koch Insitut (RKI) (102)**	Germany	2011	All Ages	309	NmA†, NmB, NmC, NmW, NmX†, NmY, Other†
**Robert Koch Insitut (RKI) (103)**	Germany	2012	All Ages	307	NmA, NmB, NmC, NmW, NmX†, NmY, Other†
**Robert Koch Insitut (RKI) (104)**	Germany	2013	All Ages	289	NmA, NmB, NmC, NmW, NmX†, NmY, Other†
**Robert Koch Insitut (RKI) (105)**	Germany	2014	All Ages	240	NmA†, NmB, NmC, NmW, NmX†, NmY, Other†
**Robert Koch Insitut (RKI) (106)***	Germany	2015	All Ages	251	NmA, NmB, NmC, NmW, NmX, NmY, Other
**Robert Koch Insitut (RKI) (107)**	Germany	2016	All Ages	280	NmA, NmB, NmC, NmW, NmX†, NmY, Other
**Theano G (108)***	Greece	2010–2016	All Ages	2010: 43<br>2011: 48<br>2012: 53<br>2013: 50<br>2014: 55<br>2015: 50<br>2016: 31	NmA, NmB, NmC, NmW, NmX, NmY, Other
**Health Protection Surveillance Centre (HPSC) (109)***	Ireland	2010	All Ages	98	NmB, NmC, NmW, NmY, Other
**Health Protection Surveillance Centre (HPSC) (110)***	Ireland	2011	All Ages	88	NmB, NmC, NmW, NmY, Other
**Health Protection Surveillance Centre (HPSC) (111)***	Ireland	2012	All Ages	60	NmB, NmC, NmW, NmY, Other
**Health Protection Surveillance Centre (HPSC) (112)***	Ireland	2013	All Ages	73	NmB, NmC, NmW, NmY, Other
**Health Protection Surveillance Centre (HPSC) (113)***	Ireland	2014	All Ages	80	NmB, NmC, NmW, NmY, Other
**Health Protection Surveillance Centre (HPSC) (114)***	Ireland	2015	All Ages	67	NmB, NmC, NmW, NmY, Other
**Higher Institute of Health (ISS) (115)**	Italy	2011–2016	All Ages	2011: 117<br>2012: 107<br>2013: 116<br>2014: 115<br>2015: 143<br>2016: 187	NmA, NmB, NmC, NmW, NmX, NmY
**Egorova E, Otorbaeva D, Ronveaux O and Wasley A (116)***	Kyrgyzstan	Jan 2014–Jul 2015	All Ages	2014: 23<br>2015: 47	NmA, NmB, NmC

**Netherlands Reference Laboratory for Bacterial Meningitis (117)**	Netherlands	2010	All Ages	132	NmA†, NmB, NmC, NmW, NmX†, NmY, Other
**Netherlands Reference Laboratory for Bacterial Meningitis (118)**	Netherlands	2011	All Ages	90	NmA†, NmB, NmC, NmW, NmX, NmY, Other
**Netherlands Reference Laboratory for Bacterial Meningitis (119)**	Netherlands	2012	All Ages	81	NmA†, NmB, NmC, NmW, NmX†, NmY, Other
**Netherlands Reference Laboratory for Bacterial Meningitis (120)**	Netherlands	2013	All Ages	111	NmA†, NmB, NmC, NmW, NmX, NmY, Other
**Netherlands Reference Laboratory for Bacterial Meningitis (121)**	Netherlands	2014	All Ages	73	NmA†, NmB, NmC, NmW, NmX, NmY, Other
**Netherlands Reference Laboratory for Bacterial Meningitis (122)**	Netherlands	2015	All Ages	84	NmA†, NmB, NmC, NmW, NmX†, NmY, Other†
**Van Der Ende A, Arends A, Feller M, Keijzers W, Schurman I, Knol Met al. (123)**	Netherlands	2016	All Ages	154	NmA†, NmB, NmC, NmW, NmX, NmY, Other†
**Blystad H, Caugant DA, Haugen IL, Rønning K, Steens A, Steinbakk Met al. (124)**	Norway	2011	All Ages	37	NmB, NmC, NmW, NmY, Other†
**Blystad H, Caugant DA, Haugen IL, Konsmo K, Steens A, Steinbakk Met al. (125)**	Norway	2012	All Ages	24	NmB, NmC, NmW, NmY, Other†
**Blystad H, Caugant DA, Haugen IL, Konsmo K, Steens A, Steinbakk Met al. (126)**	Norway	2013	All Ages	25	NmB, NmC, NmW, NmY, Other
**Caugant DA, Haugen IL, Konsmo K, Nordstrand K, Steinbakk M, Storsæter Jet al. (127)**	Norway	2014	All Ages	16	NmB, NmC, NmW, NmY, Other†
**Caugant DA, Haugen IL, Konsmo K, Nordstrand K, Steinbakk M, Storsæter Jet al. (128)**	Norway	2015	All Ages	18	NmB, NmC, NmW, NmY, Other†
**Berg A, Caugant DA, Haugen IL, Konsmo K, Steinbakk M, Storsæter Jet al. (129)**	Norway	2016	All Ages	23	NmB, NmC†, NmW, NmY, Other†
**Skoczyńska A, Kuch A, Waśko I, Gołębiewska A, Ronkiewicz P, Markowska Met al. (130)**	Poland	2010–2011	All Ages	2010: 204<br>2011: 259	NmB, NmC, NmW, NmY
**Waśko I, Kuch A, Kiedrowska M, Gołębiewska A, Ronkiewicz P, Wróbel Iet al. (131)**	Poland	2015–2016	All Ages	358	NmB, NmC, NmW, NmY
**Simões MJ and Fernandes T (132)**	Portugal	2010–2014	All Ages	2010: 60<br>2011: 68<br>2012: 55<br>2013: 54<br>2014: 43	NmA, NmB, NmC, NmW, NmY, Other
**Institutul National de Sanatate Publica (INSP) (133)**	Romania	2010	All Ages	15	NmA, NmB, NmC, Other
**Institutul National de Sanatate Publica (INSP) (134)**	Romania	2015	All Ages	27	NmA, NmB, NmC
**Koroleva I, Beloshitskij G, Zakroeva I, Melnikova A, Koroleva M, Shipulin Get al. (135)***	Russian Federation	2011	All Ages	527	NmA, NmB, NmC, NmW, NmX, NmY, Other
**Koroleva I, Beloshitskiy I, Zakroeva I and Koroleva M (136)**	Russian Federation	2014	All Ages	391	NmA, NmB, NmC
**Koroleva I, Melnikova A and Koroleva M (137)**	Russian Federation	2016	All Ages	294	NmA, NmB, NmC, NmW, NmY

**Kruzlíková A (138)**	Slovakia	2015–2016	All Ages	43	NmB, NmC, NmW, NmY
**Garrido Estepa M, Manguiña Guzmán M and Cano Portero R (139)**	Spain	Oct 2009–Sep 2013	All Ages	Oct 2009–Sep 2010: 425<br>Oct 2010–Sep 2011: 412<br>Oct 2011–Sep 2012: 356<br>Oct 2012–Sep 2013: 260	NmA, NmB, NmC, NmW, NmY, Other
**National Center of Epidemiology: Health Institute Carlos III (ISCIII) (140)**	Spain	Oct 2013–Sep 2014	All Ages	149	NmA, NmB, NmC, NmW, NmY, Other
**National Center of Epidemiology: Health Institute Carlos III (ISCIII) (141)**	Spain	Oct 2014–Aug 2016	All Ages	Oct 2014–Sep 2015: 189<br>Oct 2015–Aug 2016: 163	NmA, NmB, NmC, NmW, NmY, Other
**Public Health Agency Sweden (142)**	Sweden	2010–2016	All Ages	2010: 65<br>2011: 62<br>2012: 99<br>2013: 72<br>2014: 45<br>2015: 48<br>2016: 56	2010–2013, 2016: NmA†, NmB, NmC, NmW, NmY, Other†<br>2014, 2015: NmA†, NmB, NmC, NmW, NmY, Other
**Ninet B and Schrenzel J (143)**	Switzerland	2010	All Ages	39	NmA†, NmB, NmC, NmW, NmY
**Emonet S and Schrenzel J (144)**	Switzerland	2011	All Ages	60	NmA†, NmB, NmC, NmW, NmX†, NmY, Other
**Hinrikson H, Emonet S and Schrenzel J (145)**	Switzerland	2012	All Ages	34	NmA, NmB, NmC, NmW, NmX, NmY, Other
**Hinrikson H, Emonet S and Schrenzel J (146)**	Switzerland	2013	All Ages	42	NmA†, NmB, NmC, NmW, NmX, NmY, Other†
**Hinrikson H, Emonet S and Schrenzel J (147)**	Switzerland	2014	All Ages	35	NmA†, NmB, NmC, NmW, NmX†, NmY, Other†
**Hinrikson H, Emonet S and Schrenzel J (148)**	Switzerland	2015	All Ages	40	NmA†, NmB, NmC, NmW, NmX, NmY, Other†
**Ceyhan M, Gürler N, Ozsurekci Y, Keser M, Aycan AE, Gurbuz Vet al. (149)**	Turkey	2011–2012	<18 yrs	46	NmA, NmB, NmC, NmW, NmY, Other
**Ceyhan M, Ozsurekci Y, Gürler N, Karadag Oncel E, Camcioglu Y, Salman Net al. (150)**	Turkey	2013, 2014	<18 yrs	2013: 19<br>2014: 66	NmA, NmB, NmC, NmW, NmY, Other
**Ladhani SN, Flood JS, Ramsay ME, Campbell H, Gray SJ, Kaczmarski EBet al. (151)**	United Kingdom (England and Wales)	Jul 2009–Jun 2011	All Ages	Jul 2009–Jun 2010: 886<br>Jul 2010–Jun 2011: 1024	NmA, NmB, NmC, NmW, NmX, NmY, Other
**Public Health England (152)**	United Kingdom (England and Wales)	Jul 2011–Jun 2012	All Ages	759	NmA, NmB, NmC, NmW, NmX, NmY, Other
**Public Health England (153)**	United Kingdom (England)	Jul 2012–Jun 2013	All Ages	766	NmA, NmB, NmC, NmW, NmX, NmY, Other
**Public Health England (154)**	United Kingdom (England)	Jul 2013–Jun 2014	All Ages	630	NmA, NmB, NmC, NmW, NmX, NmY, Other
**Public Health England (155)**	United Kingdom (England)	Jul 2014–Jun 2015<br>Jul 2015–Jun 2016	All Ages	Jul 2014–Jun 2015: 720<br>Jul 2015–Jun 2016: 799	NmA, NmB, NmC, NmW, NmX, NmY, Other
**Public Health England (156)**<br>**Public Health England (157)**	United Kingdom (England)	Jul 2015–Dec 2016	All Ages	331	NmA, NmB, NmC, NmW, NmX, NmY, Other
**Public Health Wales (158)**	United Kingdom (Wales)	2013–2016	All Ages	2013: 53<br>2014: 46<br>2015: 39<br>2016: 43	NmB, NmC, NmW, NmY
**Public Health Agency (PHA) (159)***	United Kingdom (Northern Ireland)	2010–2016	All Ages	2010: 37<br>2011: 42<br>2012: 33<br>2013: 34<br>2014: 29<br>2015: 28	NmB, NmC, NmW, NmY, Other

				2016: 20	
**McDonald E, Denham B, McMenamin J and Cameron C (160)***	United Kingdom (Scotland)	2010	All Ages	42	NmB, NmC, NmW, NmY, Other
**McDonald E, Denham B, Smith-Palmer A and McMenamin J (161)**	United Kingdom (Scotland)	2011	All Ages	54	NmB, NmC, NmW, NmY, Other[†]
**Wissmann BV, Denham B, Smith-Palmer A and Cameron C (162)**	United Kingdom (Scotland)	2012	All Ages	52	NmB, NmC, NmW, NmY, Other[†]
**The Vaccine Preventable Diseases Team and the Scottish *Haemophilus Legionella* Meningococcus and Pneumococcus Reference Laboratory (SHLMPRL) (163)**	United Kingdom (Scotland)	2013	All Ages	47	NmB, NmC, NmW, NmY, Other[†]
**The Vaccine Preventable Diseases Team and the Scottish *Haemophilus Legionella* Meningococcus and Pneumococcus Reference Laboratory (SHLMPRL) (164)**	United Kingdom (Scotland)	2014	All Ages	61	NmB, NmC, NmW, NmY, Other[†]
**The Vaccine Preventable Diseases Team and the Scottish *Haemophilus Legionella* Meningococcus and Pneumococcus Reference Laboratory (SHLMPRL) (165)**	United Kingdom (Scotland)	2015	All Ages	73	NmB, NmC, NmW, NmY, Other[†]
**Health Protection Scotland Immunisation team and the Scottish *Haemophilus Legionella* Meningococcus and Pneumococcus Reference Laboratory (SHLMPRL) (166)**	United Kingdom (Scotland)	Jan–Sep 2016	All Ages	72	NmB, NmC, NmW, NmY, Other
**European Centre for Disease Prevention and Control (ECDC) (167)**	Hungary, Italy, Lithuania, Norway, Poland, Portugal, Romania, Slovakia	Hungary: 2010–2015<br>Italy: 2010<br>Lithuania: 2010–2015<br>Norway: 2010<br>Poland: 2012–2014<br>Portugal: 2015<br>Romania: 2011–2014<br>Slovakia: 2010–2012	All Ages	Hungary, 2010: 33<br>Hungary, 2011: 62<br>Hungary, 2012: 51<br>Hungary, 2013: 45<br>Hungary, 2014: 29<br>Hungary, 2015: 34<br>Italy, 2010: 110<br>Lithuania, 2010: 16<br>Lithuania, 2011: 32<br>Lithuania, 2012: 53<br>Lithuania, 2013: 76<br>Lithuania, 2014: 53<br>Lithuania, 2015: 55<br>Norway, 2010: 39<br>Poland, 2012: 225<br>Poland, 2013: 195<br>Poland, 2014: 130<br>Portugal, 2015: 60<br>Romania, 2011: 36<br>Romania, 2012: 32<br>Romania, 2013: 37<br>Romania, 2014: 40<br>Slovakia, 2010: 31<br>Slovakia, 2011: 15<br>Slovakia, 2012: 29	NmA, NmB, NmC, NmW, NmX, NmY, Other<br>2012: NmA, NmB, NmC, NmW, NmY, Other
**South East Asia Region (SEARO)**					
**Majumdar T, Bhattacharya S, Barman D and Baidya S (168)**	India	2010	All Ages	71	NmA, NmC[†], Other[†]
**Western Pacific Region (WPRO)**					

**NNDSS Annual Report Writing Group (169)**	Australia	2010	All Ages	207	NmA†, NmB, NmC, NmW, NmY
**Lahra MM and Enriquez RP (170)**	Australia	2011	All Ages	219	NmA†, NmB, NmC, NmW, NmY, Other
**Lahra MM and Enriquez RP (171)**	Australia	2012	All Ages	201	NmA†, NmB, NmC, NmW, NmY, Other
**Lahra MM and Enriquez RP (172)**	Australia	2013	All Ages	142	NmA†, NmB, NmC, NmW, NmY, Other
**Lahra MM and Enriquez RP (173)**	Australia	2014	All Ages	161	NmA†, NmB, NmC, NmW, NmY, Other
**Lahra MM, Enriquez RP and National Neisseria Network (174)**	Australia	2015	All Ages	172	NmA†, NmB, NmC, NmW, NmY
**Lahra MM and Enriquez RP (175)**	Australia	Jan–Sep 2016	All Ages	155	NmA, NmB, NmC, NmW, NmY
**Li J-h, Li Y-x, Wu D, Ning G-j, Shao Z-J and Yin Z-d (176)***	China	2010–2014	All Ages	2010: 74<br>2011: 70<br>2012: 61<br>2013: 74<br>2014: 49	NmA, NmB, NmC, NmW, NmY, Other
**National Institute of Infectious Diseases (NIID) (177)**	Japan	2013–2014	All Ages	41	NmB, NmC, NmW, NmY, Other
**Lopez L, Sexton K and Carter P (178)**	New Zealand	2010	All Ages	81	NmA†, NmB, NmC, NmW, NmX†, NmY, Other
**Lopez L, Sexton K and Carter P (179)**	New Zealand	2011	All Ages	100	NmA†, NmB, NmC, NmW, NmX†, NmY, Other
**Lopez L and Sexton K (180)**	New Zealand	2012	All Ages	68	NmA†, NmB, NmC, NmW, NmX†, NmY, Other†
**Lopez L and Sherwood J (181)**	New Zealand	2013	All Ages	57	NmA†, NmB, NmC, NmW, NmX†, NmY, Other
**Institute of Environmental Science and Research Ltd (ESR) (182)**	New Zealand	2014	All Ages	36	NmA†, NmB, NmC, NmW, NmX†, NmY, Other
**Institute of Environmental Science and Research Ltd (ESR) (183)**	New Zealand	2015	All Ages	59	NmA†, NmB, NmC, NmW, NmX†, NmY, Other†
**Institute of Environmental Science and Research Ltd (ESR) (184)**	New Zealand	2016	All Ages	67	NmA†, NmB, NmC, NmW, NmX†, NmY, Other

Visual Representation of Serogroup Distribution Estimates by WHO Region

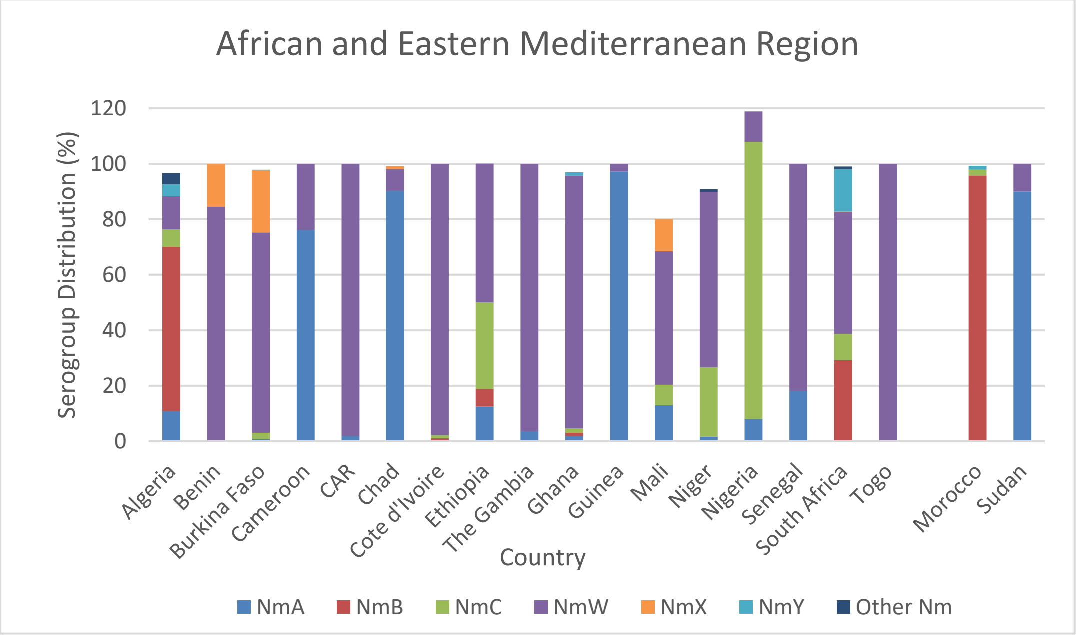

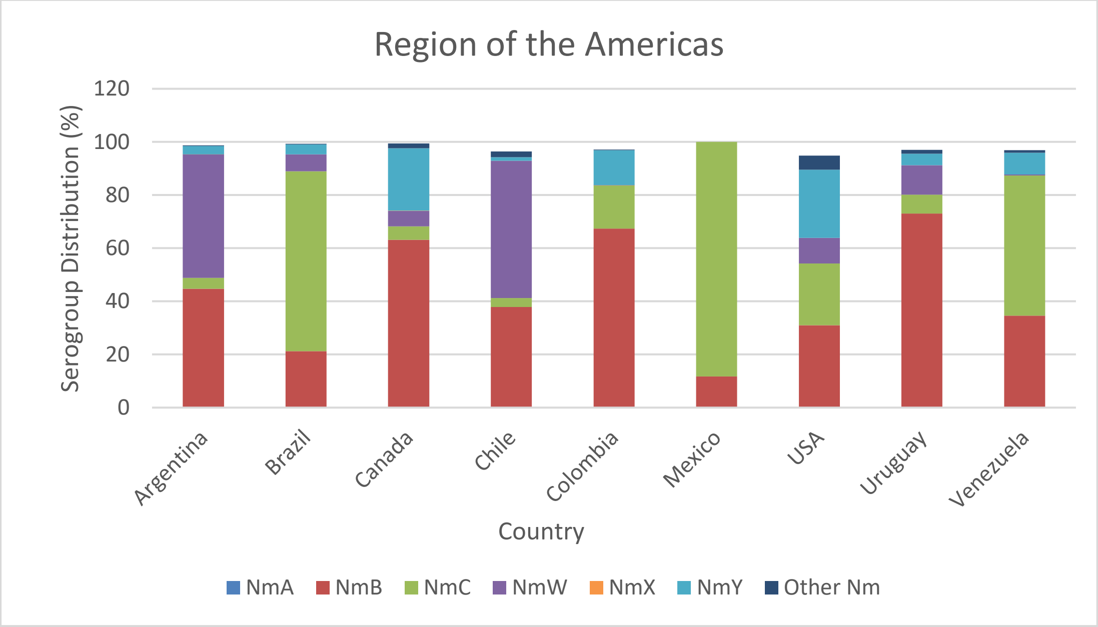

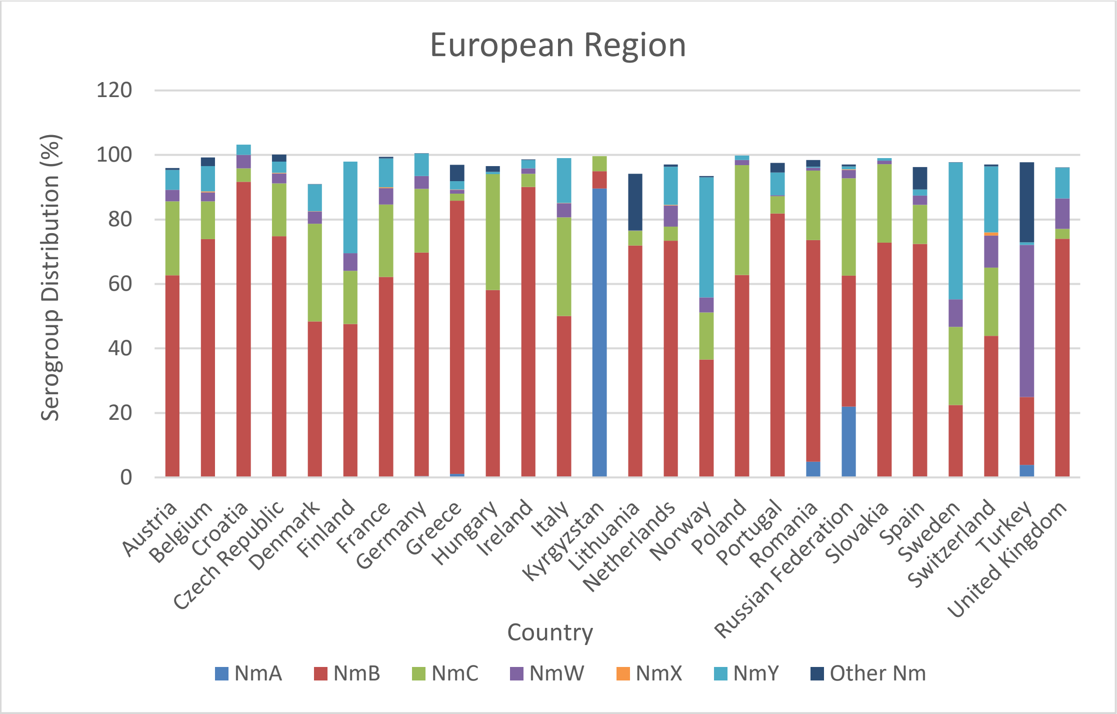
European Region
Serogroup Distribution (%)
Country
Austria
Belgium
Croatia
Czech Republic
Denmark
Finland
France
Germany
Greece
Hungary
Ireland
Italy
Kyrgyzstan
Lithuania
Netherlands
Norway
Poland
Portugal
Romania
Russian Federation
Slovakia
Spain
Sweden
Switzerland
Turkey
United Kingdom
NmA
NmB
NmC
NmW
NmX
NmY
Other Nm

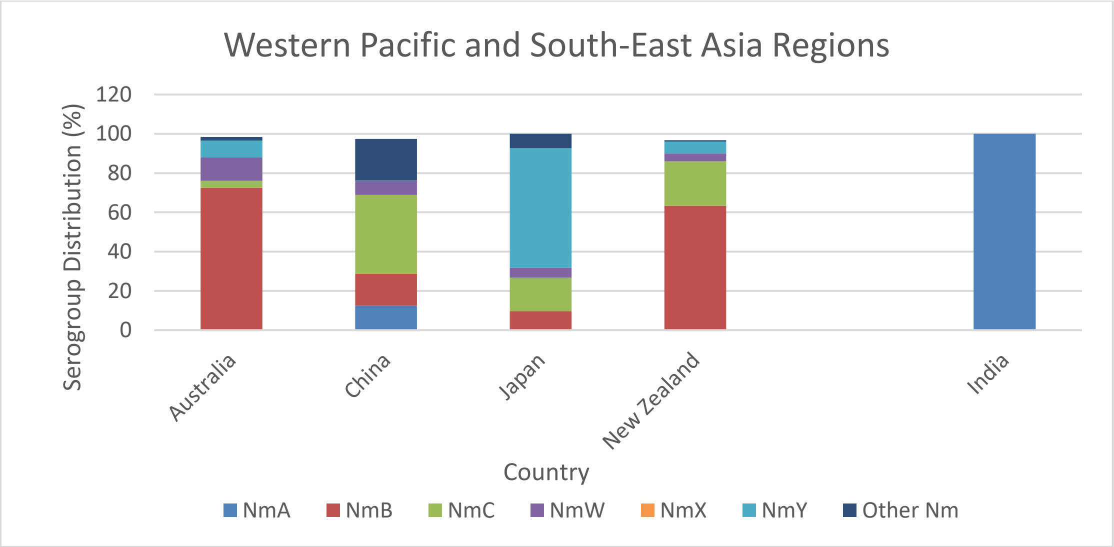
Western Pacific and South-East Asia Regions
Serogroup Distribution (%)
Country
Australia
China
Japan
New Zealand
India
NmA
NmB
NmC
NmW
NmX
NmY
Other Nm

Figure S1. Visual Representation of Included Data for the African and Eastern Mediterranean WHO Regions.

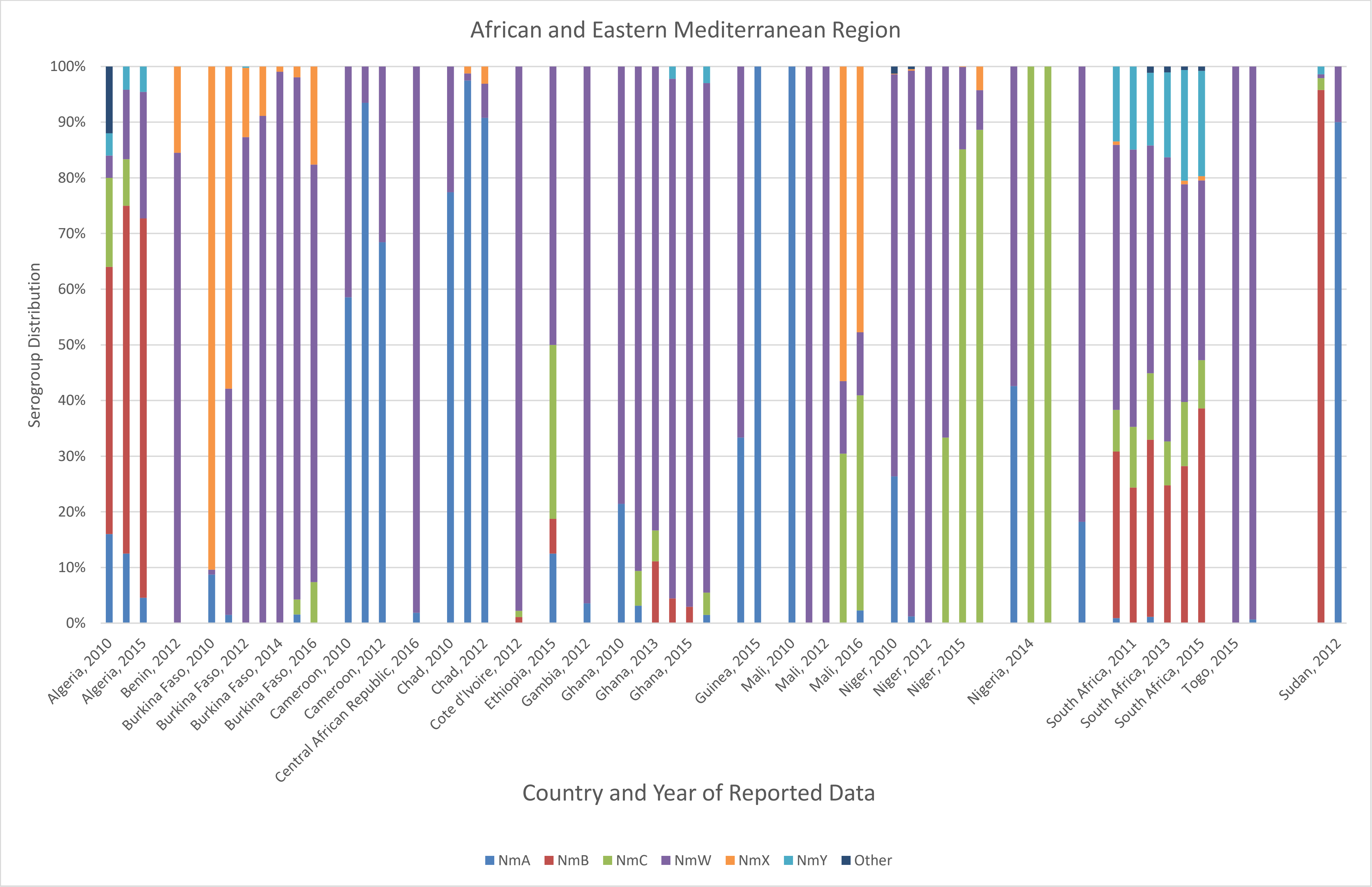

Figure S2. Visual Representation of Included Data for the WHO Region of the Americas.

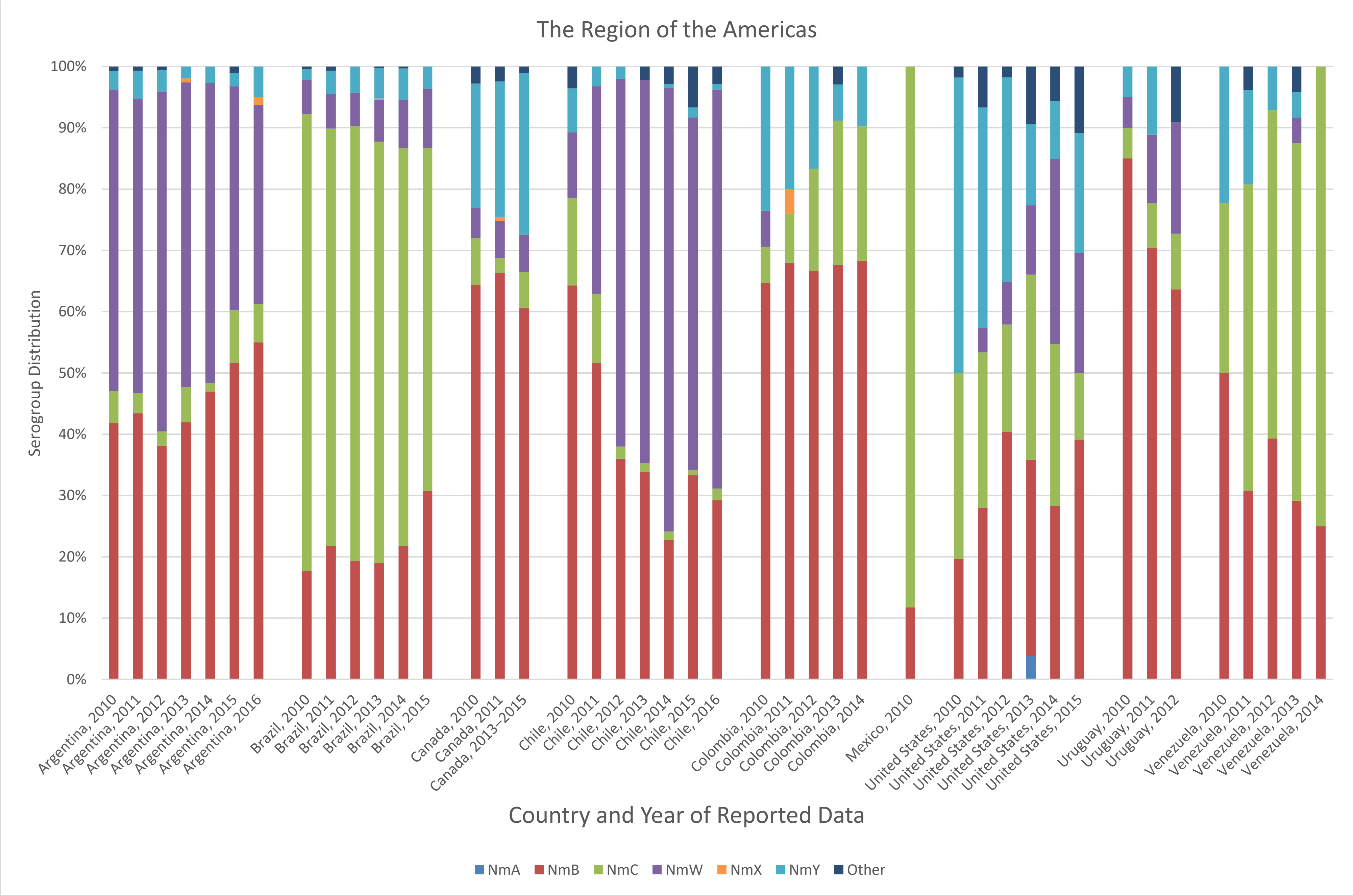

Figure S3. Visual Representation of Included Data for the European WHO Region.

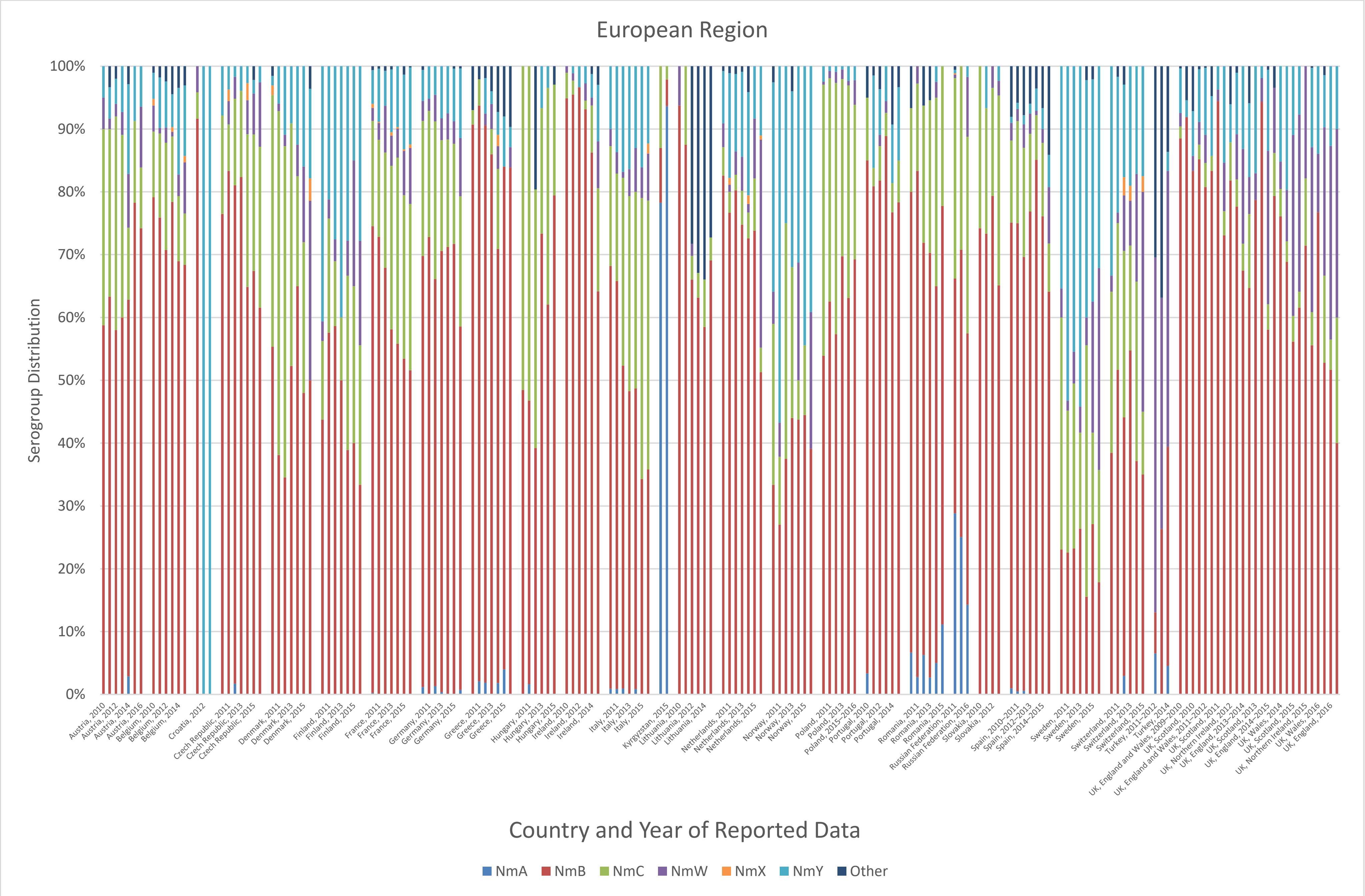

Figure S4. Visual Representation of Included Data for the Western Pacific and South-East Asia Regions.

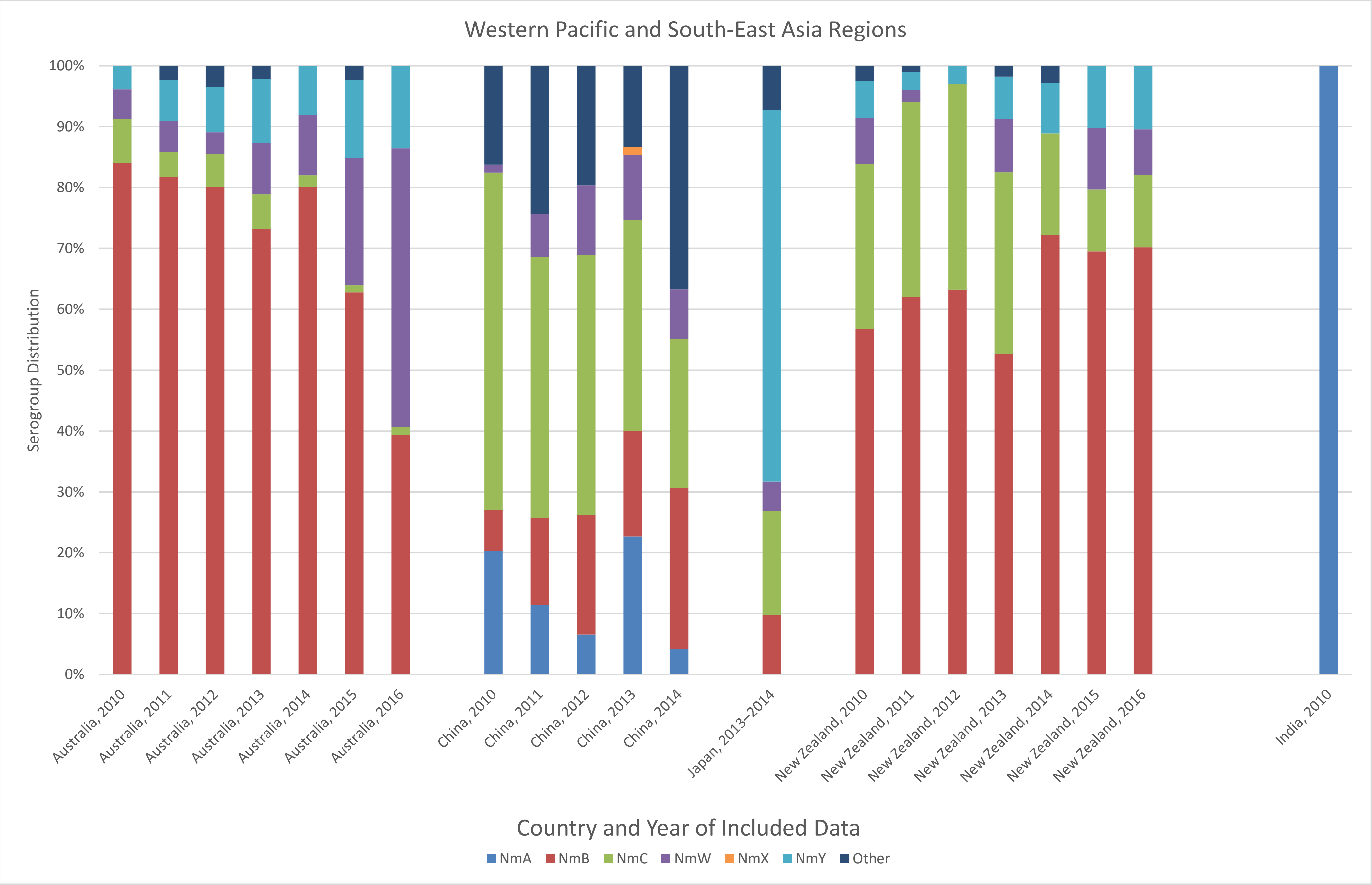

Table S4. Meta-analysis Heterogeneity.

Country	NmA p-value	NmA I^2	NmB p-value	NmB I^2	NmC p-value	NmC I^2	NmW p-value	NmW I^2	NmX p-value	NmX I^2	NmY p-value	NmY I^2	Other Nm p-value	Other Nm I^2
**African (AFRO)**														
**Algeria**	0·47	0·00%	0·37	0·37%	0·08	60·98%	0·17	43·46%	—	—	0·99	0·00%	—	—
**Benin**	—	—	—	—	—	—	—	—	—	—	—	—	—	—
**Burkina Faso**	<0·01	92·66%	—	—	<0·01	96·18%	<0·01	99·56%	<0·01	99·43%	0·79	0·00%	—	—
**Cameroon**	<0·01	91·82%	0·88	0·00%	0·88	0·00%	<0·01	91·82%	0·88	0·00%	0·88	0·00%	—	—
**Central African Republic**	—	—	—	—	—	—	—	—	—	—	—	—	—	—
**Chad**	<0·01	90·23%	—	—	—	—	<0·01	92·18%	0·33	10·33%	—	—	—	—
**Cote d'Ivoire**	—	—	—	—	—	—	—	—	—	—	—	—	—	—
**Ethiopia**	—	—	—	—	—	—	—	—	—	—	—	—	—	—
**The Gambia**	—	—	—	—	—	—	—	—	—	—	—	—	—	—
**Ghana**	0·01	68·48%	0·02	63·20%	0·24	25·42%	0·22	27·99%	0·96	0·00%	0·79	0·00%	—	—
**Guinea**	—	—	—	—	—	—	—	—	—	—	—	—	—	—
**Mali**	<0·01	98·34%	0·99	0·00%	<0·01	93·83%	<0·01	98·88%	<0·01	96·06%	0·99	0·00%	—	—
**Niger**	<0·01	99·26%	0·90	0·00%	<0·01	99·88%	<0·01	99·76%	<0·01	86·94%	0·90	0·00%	—	—
**Nigeria**	<0·01	96·53%	—	—	—	—	<0·01	97·73%	—	—	—	—	—	—
**Senegal**	—	—	—	—	—	—	—	—	—	—	—	—	—	—
**South Africa**	0·26	22·51%	0·06	53·75%	0·43	0·00%	<0·01	72·52%	0·47	0·00%	0·40	3·15%	0·98	0·00%
**Togo**	—	—	—	—	—	—	—	—	—	—	—	—	—	—
**The Americas (AMRO)**														
**Argentina**	1·00	0·00%	0·15	36·56%	0·07	47·87%	0·01	64·51%	0·73	0·00%	0·83	0·00%	0·75	0·00%
**Brazil**	1·00	0·00%	<0·01	71·27%	<0·01	82·96%	0·27	22·34%	0·83	0·00%	0·02	63·08%	0·31	15·46%
**Canada**	0·97	0·00%	0·48	0·00%	0·09	57·89%	0·88	0·00%	0·41	0·00%	0·34	7·55%	0·35	4·19%
**Chile**	1·00	0·00%	<0·01	84·48%	<0·01	74·13%	<0·01	93·46%	1·00	0·00%	0·07	48·58%	0·03	56·34%
**Colombia**	1·00	0·00%	1·00	0·00%	0·34	10·77%	0·63	0·00%	0·73	0·00%	0·31	16·69%	0·83	0·00%
**Mexico**	—	—	—	—	—	—	—	—	—	—	—	—	—	—
**United States**	0·53	0·00%	0·18	34·84%	0·11	44·09%	<0·01	86·65%	—	—	<0·01	84·74%	0·23	27·11%
**Uruguay**	0·99	0·00%	0·29	18·65%	0·91	0·00%	0·46	0·00%	0·99	0·00%	0·20	38·61%	0·19	40·06%
**Venezuela**	1·00	0·00%	0·56	0·00%	0·09	49·67%	0·82	0·00%	1·00	0·00%	0·14	41·73%	0·73	0·00%
**Eastern Mediterranean (EMRO)**														
**Morocco**	—	—	—	—	—	—	—	—	—	—	—	—	—	—
**Sudan**	—	—	—	—	—	—	—	—	—	—	—	—	—	—
**European (EURO)**														
**Austria**	0·87	0·00%	0·49	0·00%	0·03	58·06%	0·43	0·00%	1·00	0·00%	0·70	0·00%	0·51	0·00%
**Belgium**	1·00	0·00%	0·28	19·69%	0·43	0·00%	0·05	55·32%	0·69	0·00%	0·15	38·64%	0·73	0·00%
**Croatia**	—	—	—	—	—	—	—	—	—	—	0·24	29·00%	—	—
**Czech Republic**	0·96	0·00%	0·10	44·10%	0·18	32·38%	0·07	49·10%	0·79	0·00%	0·86	0·00%	—	—
**Denmark**	1·00	0·00%	0·03	55·99%	<0·01	90·25%	<0·01	79·04%	0·73	0·00%	0·09	45·40%	0·73	0·00%
**Finland**	1·00	0·00%	0·52	0·00%	0·61	0·00%	0·17	34·40%	—	—	0·43	0·00%	—	—
**France**	0·94	0·00%	<0·01	94·84%	<0·01	89·02%	<0·01	85·41%	0·39	5·26%	<0·01	79·68%	0·14	37·83%

Country	NmA p-value	NmA I^2	NmB p-value	NmB I^2	NmC p-value	NmC I^2	NmW p-value	NmW I^2	NmX p-value	NmX I^2	NmY p-value	NmY I^2	Other Nm p-value	Other Nm I^2
**Germany**	0·07	47·69%	<0·01	70·94%	0·12	40·98%	0·01	67·23%	1·00	0·00%	0·02	59·10%	0·56	0·00%
**Greece**	0·70	0·00%	0·06	50·96%	0·02	60·84%	0·43	0·00%	0·96	0·00%	0·19	31·08%	0·57	0·00%
**Hungary**	0·96	0·00%	<0·01	78·91%	<0·01	77·29%	1·00	0·00%	1·00	0·00%	0·19	32·73%	<0·01	79·52%
**Ireland**	—	—	<0·01	87·30%	<0·01	76·57%	0·17	36·11%	—	—	0·03	60·53%	0·42	0·08%
**Italy**	0·57	0·00%	<0·01	88·75%	<0·01	85·99%	0·13	39·31%	—	—	0·70	0·00%	—	—
**Kyrgyzstan**	—	—	—	—	—	—	—	—	—	—	—	—	—	—
**Lithuania**	1·00	0·00%	0·01	68·10%	0·48	0·00%	0·48	0·00%	0·99	0·00%	1·00	0·00%	<0·01	87·59%
**Netherlands**	1·00	0·00%	<0·01	85·30%	0·75	0·00%	<0·01	93·22%	0·72	0·00%	0·39	4·27%	0·26	22·50%
**Norway**	—	—	0·79	0·00%	<0·01	70·70%	0·02	59·67%	—	—	0·19	31·67%	0·89	0·00%
**Poland**	0·98	0·00%	<0·01	75·13%	<0·01	82·69%	0·10	46·48%	—	—	0·35	9·81%	0·98	0·00%
**Portugal**	0·59	0·00%	0·67	0·00%	0·69	0·00%	0·72	0·00%	—	—	<0·01	70·98%	0·17	35·36%
**Romania**	0·79	0·00%	0·53	0·00%	0·64	0·00%	0·62	0·00%	1·00	0·00%	0·72	0·00%	0·18	35·55%
**Russian Federation**	<0·01	91·66%	0·16	45·17%	0·26	25·02%	—	—	—	—	—	—	—	—
**Slovakia**	0·97	0·00%	0·63	0·00%	0·66	0·00%	0·74	0·00%	—	—	0·46	0·00%	0·97	0·00%
**Spain**	0·45	0·00%	<0·01	71·07%	<0·01	70·16%	<0·01	63·84%	—	—	0·04	54·75%	0·03	56·58%
**Sweden**	1·00	0·00%	0·79	0·00%	0·01	66·26%	<0·01	84·36%	—	—	0·06	50·78%	0·66	0·00%
**Switzerland**	0·84	0·00%	0·33	13·60%	0·29	19·38%	<0·01	81·87%	0·53	0·00%	0·50	0·00%	0·72	0·00%
**Turkey**	0·48	0·00%	<0·01	86·03%	0·92	0·00%	0·27	23·08%	—	—	0·44	0·00%	0·03	70·98%
**United Kingdom**	0·90	0·00%	<0·01	95·88%	<0·01	63·05%	<0·01	96·29%	1·00	0·00%	<0·01	63·11%	<0·01	56·35%
**South-East Asian (SEARO)**														
**India**	—	—	—	—	—	—	—	—	—	—	—	—	—	—
**Western Pacific (WPRO)**														
**Australia**	1·00	0·00%	<0·01	94·84%	0·01	64·90%	<0·01	96·10%	—	—	0·01	64·54%	0·05	58·19%
**China**	<0·01	73·24%	0·04	60·95%	0·01	69·68%	0·07	53·18%	0·84	0·00%	1·00	0·00%	0·04	61·15%
**Japan**	—	—	—	—	—	—	—	—	—	—	—	—	—	—
**New Zealand**	1·00	0·00%	0·27	21·50%	<0·01	73·74%	<0·01	68·59%	1·00	0·00%	0·32	13·98%	0·56	0·00%

Table S5. Studies not considered for inclusion in analysis based upon sample size <15 (per year average). Studies marked with an (*) were included in the analysis for other countries/time periods.

African Region (AFRO)		
**Study**	**Country**	**Year(s)**
**Réseau Algérien de Surveillance de la Résistance des Bactéries aux Antibiotiques (AARN) (185)**	Algeria	2012, 2013
**Réseau Algérien de Surveillance de la Résistance des Bactéries aux Antibiotiques (AARN) (186)**	Algeria	2014
**Nambei WS, Gamba EP, Gbangbangai E, Ouambita RM, Dalengat-Vogbia Z, Nana Ret al. (187)**	Central African Republic	2012
**Dalecha D (188)**	Ethiopia	2011
**Mihret W, Lema T (29)**	Ethiopia	2012, 2013
**Traore FA, Sako FB, Sylla D, Kader DS, Bangoura M, Traore Met al. (189)**	Guinea	2013
**Ba ID, Deme-Ly I (24)**	Senegal	2010, 2011, 2012
**Caugant DA, Kristiansen PA, Wang X, Mayer LW, Taha MK, Ouedraogo Ret al. (190)**	Uganda	2010
**Intercountry Support Team—West Africa (4)***	Code d'Ivoire, Democratic Republic of the Congo, Togo	2010
**Intercountry Support Team—West Africa (5)***	Ghana, Nigeria, Togo	2011
**Intercountry Support Team—West Africa (15)***	Benin, Central African Republic, Democratic Republic of the Congo, Nigeria, Togo	2012
**Intercountry Support Team—West Africa (9)***	Benin, Cameroon, Chad, Cote d'Ivoire, Democratic Republic of the Congo, The Gambia, Mali, Mauritania, Niger, Nigeria, Senegal, South Sudan, Sudan, Togo	2013
**Intercountry Support Team—West Africa (10)***	Benin, Democratic Republic of the Congo, The Gambia, Guinea, Mali, Senegal, Togo	2014
**Intercountry Support Team—West Africa (18)***	Benin, Cameroon, Chad, Cote d'Ivoire, The Gambia, Mauritania, Senegal	2015
**Intercountry Support Team—West Africa (19)***	Benin, Cameroon, Central African Republic, Chad, Cote d'Ivoire, Guinea	2016
**Region of the Americas (AMRO)**		
**Study**	**Country**	**Year(s)**
**Tsang RSW, Law DKS, Gad RR, Mailman T, German G and Needle R (191)**	Canada	2012
**Chanto G (192)**	Costa Rica	2010–2015
**Chacon-Cruz E, Martinez-Longoria CA, Llausas-Magana E, Luevanos-Velazquez A, Vazquez-Narvaez JA, Beltran Set al. (193)**	Mexico	2010–2013
**Cabrera-Gaytan DA, Perez-Perez GF, Arriaga-Nieto L, Vallejos-Paras A, Padilla-Velazquez R and Grajales-Muniz C (194)**	Mexico	2012–2014
**Pan American Health Organization (PAHO) (51)***	Costa Rica, Cuba, Dominican Republic, Ecuador, El Salvador, Nicaragua, Panama, Paraguay, Peru	2010
**Pan American Health Organization (PAHO) (52)***	Costa Rica, Cuba, Dominican Republic, Ecuador, El Salvador, Guatemala, Honduras, Mexico, Panama, Paraguay	2011
**Pan American Health Organization (PAHO) (53)***	Bolivia, Costa Rica, Cuba, Dominican Republic, Ecuador, El Salvador, Honduras, Mexico, Nicaragua, Paraguay, Peru	2012
**Pan American Health Organization (PAHO) (54)***	Bolivia, Costa Rica, Cuba, Dominican Republic, Ecuador, El Salvador, Mexico, Nicaragua, Panama, Paraguay, Peru, Uruguay	2013
**Pan American Health Organization (PAHO) (55)***	Bolivia, Costa Rica, Cuba, Dominican Republic, El Salvador, Mexico, Paraguay, Uruguay	2014
**Eastern Mediterranean Region (EMRO)**		
**Study**	**Country**	**Year(s)**
**Husain EH, Barakat M and Al-Saleh M (195)**	Kuwait	2010–2013
**Intercountry Support Team—West Africa (9)***	Sudan	2013
**European Region (EURO)**		
**Study**	**Country**	**Year(s)**

**Bukovski S, Vacca P, Anselmo A, Knezovic I, Fazio C, Neri Aet al. (196)**	Croatia	2009–2014
**Klismanic Z, Juretic KB and Tripkovic I (197)**	Croatia	2010
**Bukovski S, Švagelj A and Kaić B (198)**<br>*Although this study has an average of >15 cases per year, there were <15 in 2016 and in 2017. Therefore, it was not included in the analysis.*	Croatia	2015–2017
**Eloshvili M, McHedlishvili I and Imnadze P (199)**	Georgia	2009–2011
**Infectious Disease Prevention and Control Unit (200)**	Malta	2010–2012
**National Reference Laboratory for Meningococcus and Haemophilus (NRLMH) (201)**	Serbia	2009–2014
**European Centre for Disease Prevention and Control (ECDC) (167)***	Bulgaria, Cyprus, Estonia, Iceland, Latvia, Luxembourg, Malta, Slovakia, Slovenia	Bulgaria: 2015; Cyprus: 2010–2015; Estonia: 2010–2012; Iceland: 2010–2015; Latvia: 2010–2015; Luxembourg: 2010, 2014; Malta: 2013–2015; Slovakia: 2013, 2014; Slovenia: 2010–2015
**South-East Asian Region (SEARO)**		
**Study**	**Country**	**Year(s)**
**Majumdar T, Bhattacharya S (168)***	India	2011, 2012
**Western Pacific Region (WPRO)**		
**Study**	**Country**	**Year(s)**
**National Institute of Infectious Diseases (NIID) (202)**	Japan	2015

Table S6. Antibiotics Tested for antimicrobial susceptibility.

Antibiotics Tested for Antimicrobial Susceptibility	Algeria	Burkina Faso	Cameroon	CAR	Cote d'Ivoire	DRC	Eritrea	Ethiopia	Ghana	Kenya	Madagascar	Mali	Mauritania	Mozambique	Niger	Rwanda	Senegal	Tanzania	Togo	Uganda	Sudan	Azerbaijan	Bulgaria	Czech Republic	Denmark	Estonia	Georgia	Greece	Ireland	Latvia	Luxembourg	Malta	Norway	Poland	Portugal	Scotland	Slovakia	Spain	Sweden	Bangladesh	Nepal	Sri Lanka	Cambodia	Mongolia	Philippines
Penicillins																																													
Amoxicillin			✓		✓						✓																✓																		
Amoxicillin + clavulanate		✓														✓			✓																									✓	
Ampicillin	✓	✓		✓§		✓		✓	✓	✓				✓	✓	✓											✓		✓	✓	✓							✓						✓	✓
Penicillin G/ BenzylPenicillin/ Penicillin	✓	✓	✓	✓§	✓			✓			✓				✓	✓		✓	✓				✓	✓	✓	✓	✓	✓	✓	✓	✓	✓	✓	✓	✓	✓	✓	✓	✓		✓	✓	✓		✓
Penicillin on oxacillin disk																																												✓	
Oxacillin			✓	✓§	✓	✓						✓				✓			✓																										
Second Generation Cephalosporins																																													
Cefaclor																												✓																	
Cefuroxime										✓																																			
Cefoxitin																																									✓				
Third Generation Cephalosporins																																													
Cefixime																							✓																						
Ceftazidime										✓																																		§	
Cefotaxime	§		§	✓§		✓		*		✓	✓					✓		✓		✓						✓	✓	✓	✓			✓		✓		✓	✓	✓	✓		✓	§		§	✓§
Ceftriaxone	§	✓	§	✓§	✓	✓	✓	*	✓	✓		✓	✓	✓	✓	✓		✓	✓	✓	✓	✓		✓	✓	✓	✓	✓		✓	✓	✓	✓		✓			✓				§	✓	§	✓§
Fourth Generation Cephalosporins																																													
Cefepime										✓																																			
Fluroquinolones																																													
Ciprofloxacin	✓	✓	✓	*			✓	*	✓		✓		✓			✓		✓		✓		✓	✓	✓	✓	*	✓	✓	✓		✓	✓	†	✓	✓	✓	✓	✓	✓		✓	✓	✓		
Ciprofloxacin + nalidixic acid																																													*
Levofloxacin								*																																					*
Nalidixic Acid				✓			✓																																						
Ofloxacin																																									✓				
Chloramphenicol																																													
Chloramphenicol	✓		✓	*	✓	✓	✓	*	✓	✓	✓	✓	✓	✓	✓				✓	✓	✓	✓	✓				✓	✓					†	✓		✓			✓		✓	✓	✓	✓	✓

Rifamycins																																													
Rifampicin	✓		✓	✓				*			✓		✓			✓		✓	✓	✓	✓	✓	✓	✓	✓	*	✓	✓	✓		✓	✓	†	✓	✓	✓	✓	✓	✓						*
Betalactamines																																													
(general)																	✓																												*
Aminoglycosides																																													
Gentamicin		✓				✓													✓																										
Carbapenems																																													
Imipenem																																												✓	
Meropenem								*		✓													✓			*	✓		✓	✓	✓								✓		✓	✓			✓
Macrolides																																													
Azithromycin	✓							*					✓									✓											†								✓				*
Erythromycin		✓														✓					✓																								
Sulfonamides																																													
Cotrimoxazole								*					✓						✓													✓		†							✓				*
Sulfisoxazole																																													*
Sulphamethoxazole																																				‡			✓						
(general)																								✓									†												
Tetracyclines																																													
Tetracycline																											✓																		
Minocycline								*																																					*
Nitroimidazoles																																													
Metronidazole		✓																																											

(✓): primary tests

(*): secondary/alternative tests as per country protocols

(†): only at national reference laboratory

(‡): will soon discontinue

(§): one of these options per drug class

References

1 Ky-Ba A, Sanou M, Tranchot JD, Christiasen PA, Ouedraogo AS, Tamboura M, et al. Dynamics of germs responsible for acute bacterial meningitis in Burkina Faso in the last ten years (2005–2014). African Journal of Clinical and Experimental Microbiology. 2016;17:10–7.

2 Delrieu I, Yaro S, Tamekloé TAS, Njanpop-Lafourcade BM, Tall H, Jaillard P, et al. Emergence of epidemic *Neisseria meningitidis* serogroup X meningitis in Togo and Burkina Faso. PLoS One. 2011;6:e19513.

3 Novak RT, Kambou JL, Diomandé FVK, Tarbangdo TF, Ouédraogo-Traoré R, Sangaré L, et al. Serogroup A meningococcal conjugate vaccination in Burkina Faso: analysis of national surveillance data. The Lancet Infectious Diseases. 2012;12:757–64.

4 Intercountry Support Team—West Africa. Meningitis weekly bulletin: week 48–52 2010. Ouagadougou, Burkina Faso: World Health Organization (WHO) Regional Office for Africa, 2011.

5 Intercountry Support Team—West Africa. Meningitis weekly bulletin: week 44–47 2011. Ouagadougou, Burkina Faso: World Health Organization (WHO) Regional Office for Africa, 2011.

6 MacNeil JR, Medah I, Koussoube D, Novak RT, Cohn AC, Diomande FVK, et al. Neisseria meningitidis serogroup W, Burkina Faso, 2012. Emerging Infectious Diseases. 2014;20:394–9.

7 Cibrelus L, Medah I, Koussoubé D, Yélbeogo D, Fernandez K, Lingani C, et al. Serogroup W meningitis outbreak at the subdistrict level, Burkina Faso, 2012. Emerging Infectious Diseases. 2015;21:2063–6.

8 Savadogo M, Kyélem N, Yelbeogo D, Koussoubé D, Tarbagdo F, Ouédraogo A. [The *Neisseria meningitidis* W135 epidemic in 2012 in Burkina Faso]. Bulletin de la Societe de Pathologie Exotique. 2014;107:15–7.

9 Intercountry Support Team—West Africa. Meningitis weekly bulletin: week 49–52 2013. Ouagadougou, Burkina Faso: World Health Organization (WHO) Regional Office for Africa, 2013.

10 Intercountry Support Team—West Africa. Meningitis weekly bulletin: week 49–52 2014. Ouagadougou, Burkina Faso: World Health Organization (WHO) Regional Office for Africa, 2014.

11 Massenet D, Birguel J, Azowé F, Ebong C, Gake B, Lombart JP, et al. Epidemiologic pattern of meningococcal meningitis in northern Cameroon in 2007-2010: contribution of PCR-enhanced surveillance. Pathogens and Global Health. 2013;107:15–20.

12 Massenet D, Vohod D, Hamadicko H, Caugant DA. Epidemic meningococcal meningitis, Cameroon. Emerging Infectious Diseases. 2011;17:2070–2.

13 Gamougam K, Daugla DM, Toralta J, Ngadoua C, Fermon F, Page AL, et al. Continuing effectiveness of serogroup A meningococcal conjugate vaccine, Chad, 2013. Emerging Infectious Diseases. 2015;21:115–8.

14 Daugla DM, Gami JP, Gamougam K, Naibei N, Mbainadji L, Narbé M, et al. Effect of a serogroup A meningococcal conjugate vaccine (PsA-TT) on serogroup A meningococcal meningitis and carriage in Chad: a community study. Lancet. 2014;383:40–7.

15 Intercountry Support Team—West Africa. Meningitis weekly bulletin: week 49–52 2012. Ouagadougou, Burkina Faso: World Health Organization (WHO) Regional Office for Africa, 2012.

16 Hossain MJ, Roca A, Mackenzie GA, Jasseh M, Hossain I, Muhammad S, et al. Epidemiology and risk factors of serogroup W135 meningococcal disease outbreak in the Gambia, February–June 2012. American Journal of Tropical Medicine and Hygiene. 2013;89:453.

17 Hossain MJ, Roca A, Mackenzie GA, Jasseh M, Hossain MI, Muhammad S, et al. Serogroup W135 meningococcal disease, The Gambia, 2012. Emerging Infectious Diseases. 2013;19:1507–10.

18 Intercountry Support Team—West Africa. Meningitis weekly bulletin: week 49–53 2015. Ouagadougou, Burkina Faso: World Health Organization (WHO) Regional Office for Africa, 2016.

19 Intercountry Support Team—West Africa. Meningitis weekly bulletin: week 48–52 2016. Ouagadougou, Burkina Faso: World Health Organization (WHO) Regional Office for Africa, 2017.

20 Kwambana-Adams BA, Asiedu-Bekoe F, Sarkodie B, Afreh OK, Kuma GK, Owusu-Okyere G, et al. An outbreak of pneumococcal meningitis among older children (>=5 years) and adults after the implementation of an infant vaccination programme with the 13-valent pneumococcal conjugate vaccine in Ghana. BMC Infectious Diseases. 2016;16:575.

21 MenAfriNet. MenAfriNet surveillance annual feedback bulletin: quarter 1. MenAfriNet, 2016.

22 Collard JM, Issaka B, Zaneidou M, Hugonnet S, Nicolas P, Taha MK, et al. Epidemiological changes in meningococcal meningitis in Niger from 2008 to 2011 and the impact of vaccination. BMC Infectious Diseases. 2013;13.

23 Ndow G, Manga NM, Ba IO, Ka D, Cisse-Diallo V, Diop SA, et al. Role of *Neisseria meningitidis* W135 in the cerebrospinal meningitis outbreak in Senegal in 2012: epidemiological and biological characteristics. International Journal of Infectious Diseases. 2014;21:148.

24 Ba ID, Deme-Ly I, Thiongane A, Diop A, Sonko A, Keita LM, et al. [Meningococcal meningitis in Senegalese pediatric setting: on purpose of 79 cases]. Medecine d'Afrique Noire. 2016;63:277–86.
25 Réseau Algérien de Surveillance de la Résistance des Bactéries aux Antibiotiques (AARN). [Bacterial antimicrobial resistance surveillance: 12th Evaluation Report]. Ministère de la Santé, de la Population et de la Réforme Hospitalière, République Algérienne Démocratique et Populaire, 2011.
26 Réseau Algérien de Surveillance de la Résistance des Bactéries aux Antibiotiques (AARN). [Bacterial antimicrobial resistance surveillance: 13th Evaluation Report]. Ministère de la Santé, de la Population et de la Réforme Hospitalière, République Algérienne Démocratique et Populaire, 2012.
27 Réseau Algérien de Surveillance de la Résistance des Bactéries aux Antibiotiques (AARN). [Bacterial antimicrobial resistance surveillance: 16th Evaluation Report]. Ministère de la Santé, de la Population et de la Réforme Hospitalière, République Algérienne Démocratique et Populaire, 2017.
28 Njanpop-Lafourcade BM, Hugonnet S, Djogbe H, Kodjo A, N'Douba AK, Taha MK, et al. Mobile microbiological laboratory support for evaluation of a meningitis epidemic in Northern Benin. PLoS One. 2013;8:e68401.
29 Mihret W, Lema T, Merid Y, Kassu A, Abebe W, Moges B, et al. Surveillance of bacterial meningitis, Ethiopia, 2012–2013. Emerging Infectious Diseases. 2016;22:75–8.
30 National Institute for Communicable Diseases (NICD). GERMS South Africa: annual report 2010. Johannesburg, South Africa: NICD Division of the National Health Laboratory Service, 2010.
31 National Institute for Communicable Diseases (NICD). GERMS South Africa: annual report 2011. Johannesburg, South Africa: NICD Division of the National Health Laboratory Service, 2011.
32 National Institute for Communicable Diseases (NICD). GERMS South Africa: annual report 2012. Johannesburg, South Africa: NICD Division of the National Health Laboratory Service, 2012.
33 National Institute for Communicable Diseases (NICD). GERMS South Africa: annual report 2013. Johannesburg, South Africa: NICD Division of the National Health Laboratory Service, 2013.
34 National Institute for Communicable Diseases (NICD). GERMS South Africa: annual report 2014. Johannesburg, South Africa: NICD Division of the National Health Laboratory Service, 2014.
35 National Institute for Communicable Diseases (NICD). GERMS South Africa: annual report 2015. Johannesburg, South Africa: NICD Division of the National Health Laboratory Service, 2016.
36 Regueira M, Corso A. [Characterization of *Neisseria meningitidis* isolates, Argentina, 2015: SIREVA II]. National Institute of Infectious Diseases (INEI-ANLIS), 2016.
37 Regueira M, Corso A, Efron A, Gagetti P. [Information about the surveillance of bacterial pneumonia and meningitis: SIREVA II. PAHO. 2016: *Neisseria meningitidis*]. National Institute of Infectious Diseases (INEI-ANLIS), 2017.
38 Silva de Lemos A, Outeiro Gorla M, Cobo Zanella R, Grassi Almeida S, Bokermann S, De Cunto Brandileone M. [Surveillance information of pneumonias and bacterial meningitis: SIREVA II, Brazil, PAHO]. São Paolo, Brazil: Instituto Adolfo Lutz (IAL), Center for Meningitis P, and Pneumococcal Infections (NMPI); 2016.
39 Li YA, Tsang R, Desai S, Deehan H. Enhanced surveillance of invasive meningococcal disease in Canada, 2006–2011. Canada Communicable Disease Report. 2014;40:160–9.
40 Tsang RS, Law DK, Deng S, Hoang L. Ciprofloxacin-resistant *Neisseria meningitidis* in Canada: likely imported strains. Canadian Journal of Microbiology. 2017;63:265–8.
41 Instituto de Salud Pública de Chile (ISP). [Report of the results of laboratory surveillance: invasive disease *Neisseria meningitidis* 2013]. Ministerio de Salud, Gobierno de Chile, 2013.
42 Instituto de Salud Pública de Chile (ISP). [Report of the results of laboratory surveillance: invasive disease *Neisseria meningitidis* 2014]. Ministerio de Salud, Gobierno de Chile, 2015.
43 Instituto de Salud Pública de Chile (ISP). [Report of the results of laboratory surveillance: invasive disease *Neisseria meningitidis* 2015]. Ministerio de Salud, Gobierno de Chile, 2016.
44 Instituto de Salud Pública de Chile (ISP). [Report of the results of laboratory surveillance: invasive disease *Neisseria meningitidis* 2016]. Ministerio de Salud, Gobierno de Chile, 2016.
45 Centers for Disease Control and Prevention (CDC). Active Bacterial Core Surveillance (ABCs) report: Emerging Infections Program Network *Neisseria meningitidis*, 2010. CDC, 2012.
46 Centers for Disease Control and Prevention (CDC). Active Bacterial Core Surveillance (ABCs) report: Emerging Infections Program Network *Neisseria meningitidis,* 2011. CDC, 2013.
47 Centers for Disease Control and Prevention (CDC). Active Bacterial Core Surveillance (ABCs) report: Emerging Infections Program Network *Neisseria meningitidis,* 2012. CDC, 2013.
48 Centers for Disease Control and Prevention (CDC). Active Bacterial Core Surveillance (ABCs) report: Emerging Infections Program Network *Neisseria meningitidis,* 2013. CDC, 2015.

49 Centers for Disease Control and Prevention (CDC). Active Bacterial Core Surveillance (ABCs) report: Emerging Infections Program Network *Neisseria meningitidis,* 2014. CDC, 2016.
50 Centers for Disease Control and Prevention (CDC). Active Bacterial Core Surveillance (ABCs) report: Emerging Infections Program Network *Neisseria meningitidis,* 2015. CDC, 2017.
51 Pan American Health Organization (PAHO). [SIREVA II regional report, 2010: data by country and by age group on the characteristics of isolates of *Streptococcus pneumoniae*, *Haemophilus influenzae,* and *Neisseria meningitidis* in invasive processes]. Washington D.C., USA: PAHO, 2011.
52 Pan American Health Organization (PAHO). [SIREVA II regional report, 2011: data by country and by age group on the characteristics of isolates of *Streptococcus pneumoniae*, *Haemophilus influenzae,* and *Neisseria meningitidis* in invasive processes]. Washington D.C., USA: PAHO, 2012.
53 Pan American Health Organization (PAHO). [SIREVA II regional report, 2012: Data by country and by age group on the characteristics of isolates of *Streptococcus pneumoniae*, *Haemophilus influenzae,* and *Neisseria meningitidis* in invasive processes]. Washington D.C., USA: PAHO, 2013.
54 Pan American Health Organization (PAHO). [SIREVA II regional report, 2013: Data by country and by age group on the characteristics of isolates of *Streptococcus pneumoniae*, *Haemophilus influenzae,* and *Neisseria meningitidis* in invasive processes]. Washington D.C., USA: PAHO, 2016.
55 Pan American Health Organization (PAHO). [SIREVA II regional report, 2014: Data by country and by age group on the characteristics of isolates of *Streptococcus pneumoniae*, *Haemophilus influenzae,* and *Neisseria meningitidis* in invasive processes]. Washington D.C., USA: PAHO, 2016.
56 Razki A, Zerouali K, Belabbas H, Bouayad A, Elmdaghri N, Deghmane AE, et al. Invasive meningococcal disease: surveillance in Casablanca (Morocco). 14th Congress EMGM; 18–21 September 2017; Prague, Czech Republic2017. p. 99.
57 National Reference for Meningococcal Disease. [Annual report 2010]. Graz, Austria: Austrian Agency for Health and Food Security (AGES), 2011.
58 National Reference for Meningococcal Disease. [Annual report 2011]. Graz, Austria: Austrian Agency for Health and Food Security (AGES), 2012.
59 National Reference for Meningococcal Disease. [Annual report 2012]. Graz, Austria: Austrian Agency for Health and Food Security (AGES), 2013.
60 National Reference for Meningococcal Disease. [Annual report 2013]. Graz, Austria: Austrian Agency for Health and Food Security (AGES), 2014.
61 National Reference for Meningococcal Disease. [Annual report 2014]. Graz, Austria: Austrian Agency for Health and Food Security (AGES), 2015.
62 National Reference for Meningococcal Disease. [Annual report 2015]. Graz, Austria: Austrian Agency for Health and Food Security (AGES), 2016.
63 National Reference for Meningococcal Disease. [Annual report 2016]. Graz, Austria: Austrian Agency for Health and Food Security (AGES), 2017.
64 Bertrand S, Carion F, Stragier P. [National Reference Center for *Neisseria meningitidis*: annual report 2010]. Brussels, Belgium: Public Health Scientific Institute (ISP WIV), 2011.
65 Bertrand S, Mattheus W, Vanhoof R, Carion F. [National Reference Center for *Neisseria meningitidis*: annual report 2011]. Brussels, Belgium: Public Health Scientific Institute (ISP WIV), 2012.
66 Bertrand S, Mattheus W, Vanhoof R. [National Reference Center for *Neisseria meningitidis*: annual report 2012]. Brussels, Belgium: Public Health Scientific Institute (ISP WIV), 2013.
67 Bertrand S, Mattheus W, Vanhoof R. [National Reference Center for *Neisseria meningitidis*: annual report 2013]. Brussels, Belgium: Public Health Scientific Institute (ISP WIV), 2014.
68 Bertrand S, Mattheus W, Vanhoof R. [National Reference Center for *Neisseria meningitidis*: annual report 2014]. Brussels, Belgium: Public Health Scientific Institute (ISP WIV), 2015.
69 Bertrand S, Mattheus W, Vanhoof R, Ceyssens P. [National Reference Center for *Neisseria meningitidis*: annual report 2015]. Brussels, Belgium: Public Health Scientific Institute (ISP WIV), 2016.
70 Čeljuska-Tošev E, Bukovski-Simonoski S, Gužvinec M, Kovačević G, Knezović I. [Meningococcal disease: epidemiological and clinical features]. Paediatria Croatica. 2011;55:98–105.
71 Bröker M, Bukovski S, Culic D, Jacobsson S, Koliou M, Kuusi M, et al. Meningococcal serogroup Y emergence in Europe: high importance in some European regions in 2012. Human Vaccines & Immunotherapeutics. 2014;10:1725–8.
72 Bröker M, Emonet S, Fazio C, Jacobsson S, Koliou M, Kuusi M, et al. Meningococcal serogroup Y disease in Europe: continuation of high importance in some European regions in 2013. Human Vaccines & Immunotherapeutics. 2015;11:2281–6.

73 Křížová P, Musílek M, Vacková Z, Kozáková J. [Invasive meningococcal disease in the Czech Republic in 2010]. Prague, Czech Republic: National Institute of Public Health (SZÚ), 2011.
74 Křížová P, Musílek M, Vacková Z, Kozáková J. [Invasive meningococcal disease in the Czech Republic in 2011]. Prague, Czech Republic: National Institute of Public Health (SZÚ), 2012.
75 Křížová P, Musílek M, Vacková Z, Kozáková J. [Invasive Meningococcal Disease in the Czech Republic in 2012]. Prague, Czech Republic: National Institute of Public Health (SZÚ), 2013.
76 Křížová P, Musílek M, Vacková Z, Kozáková J. [Invasive meningococcal disease in the Czech Republic in 2013]. Prague, Czech Republic: National Institute of Public Health (SZÚ), 2014.
77 Křížová P, Musílek M, Vacková Z, Bečvářová Z, Kozáková J, Šebestová H. [Invasive Meningococcal Disease in the Czech Republic in 2014]. Prague, Czech Republic: National Institute of Public Health (SZÚ), 2015.
78 Křížová P, Musílek M, Vacková Z, Bečvářová Z, Kozáková J, Šebestová H. [Invasive Meningococcal Disease in the Czech Republic in 2015]. Prague, Czech Republic: National Institute of Public Health (SZÚ), 2016.
79 Křížová P, Musílek M, Vacková Z, Jandová Z, Kozáková J, Šebestová H. [Invasive Meningococcal Disease in the Czech Republic in 2016]. Prague, Czech Republic: National Institute of Public Health (SZÚ), 2017.
80 Rasmussen JN, Valentiner-Branth P, Hoffmann S, Mygh A. Meningococcal disease 2010. Copenhagen, Denmark: Statens Serum Institut (SSI), 2011.
81 Bjerre C, Valentiner-Branth P, Dalby T, Hoffmann S. Meningococcal disease 2011. Copenhagen, Denmark: Statens Serum Institut (SSI), 2012.
82 Suppli CH, Valentiner-Branth P, Hoffmann S. Meningococcal disease 2012. Copenhagen, Denmark: Statens Serum Institut (SSI), 2013.
83 Suppli CH, Valentiner-Branth P, Hoffmann S. Meningococcal disease 2013. Copenhagen, Denmark: Statens Serum Institut (SSI), 2014.
84 Voss S, Suppli CH, Valentiner-Branth P, Hoffmann S. Meningococcal disease 2014. Copenhagen, Denmark: Statens Serum Institut (SSI), 2015.
85 Espenhain L, Suppli CH, Valentiner-Branth P, Fuursted K, Hoffmann S. Meningococcal disease 2015. Copenhagen, Denmark: Statens Serum Institut (SSI), 2016.
86 Valentiner-Branth P, Andersen P, Christiansen A, Krause T, Ertner G, Nørgaard J, et al. Increase in the incidence of invasive meningococcal disease caused by group W135. Copenhagen, Denmark: Statens Serum Institut (SSI), 2017.
87 National Institute for Health and Welfare (THL). Infectious diseases in Finland 2010. Helsinki, Finland: THL Department of Infectious Disease Surveillance and Control, 2011.
88 National Institute for Health and Welfare (THL). Infectious diseases in Finland 2011. Helsinki, Finland: THL Department of Infectious Disease Surveillance and Control, 2012.
89 National Institute for Health and Welfare (THL). Infectious diseases in Finland 2012. Helsinki, Finland: THL Department of Infectious Disease Surveillance and Control, 2013.
90 National Institute for Health and Welfare (THL). Infectious diseases in Finland 2013. Helsinki, Finland: THL Department of Infectious Disease Surveillance and Control, 2014.
91 National Institute for Health and Welfare (THL). Infectious diseases in Finland 2014. Helsinki, Finland: THL Department of Infectious Disease Surveillance and Control, 2015.
92 National Institute for Health and Welfare (THL). Infectious diseases in Finland 2015. Helsinki, Finland: THL Department of Infectious Disease Surveillance and Control, 2016.
93 Toropainen M, Vainio A, Kuusi M. Epidemiology of invasive meningococcal disease in Finland, 2016. 14th Congress EMGM; 18–21 September 2017; Prague, Czech Republic2017. p. 48.
94 Châtelet IPd, Taha MK, Lepoutre A, Maine C, Deghmane AE, Lévy-Bruhl D. [Invasive meningococcal disease in France in 2010]. Bulletin Épidémiologique Hebdomadaire. 2011;2011:475–80.
95 Châtelet IPd, Taha MK, Lepoutre A, Maine C, Deghmane AE, Lévy-Bruhl D. [Invasive meningococcal disease in France in 2011: main epidemiological features]. Bulletin Épidémiologique Hebdomadaire. 2012;2012:569–73.
96 Barret AS, Deghmane AE, Lepoutre A, Fonteneau L, Maine C, Taha MK, et al. [Invasive meningococcal disease in France in 2012: main epidemiological features]. Bulletin Épidémiologique Hebdomadaire. 2014;2014:25–31.
97 Santé Publique France. [Invasive meningococcal infections in France in 2013]. 2015. Available: http://invs.santepubliquefrance.fr/fr../Dossiers-thematiques/Maladies-infectieuses/Maladies-a-prevention-vaccinale/Infections-invasives-a-meningocoques/Donnees-epidemiologiques/Les-infections-invasives-a-meningocoque-en-France-en-2013. Accessed.
98 Santé Publique France. [Invasive meningococcal infections in France in 2014]. 2016. Available: http://invs.santepubliquefrance.fr/fr../Dossiers-thematiques/Maladies-infectieuses/Maladies-a-prevention-

vaccinale/Infections-invasives-a-meningocoques/Donnees-epidemiologiques/Les-infections-invasives-a-meningocoque-en-France-en-2014. Accessed.
99 Santé Publique France. [Invasive meningococcal infections in 2015]. Institut de Veille Sanitaire (InVS), 2016.
100 Santé Publique France. [Invasive meningococcal infections in 2016]. Institut de Veille Sanitaire (InVS), 2017.
101 Robert Koch Insitut (RKI). [Epidemiology of notifiable infectious diseases: 2010 annual report]. Berlin, Germany: RKI, 2011.
102 Robert Koch Insitut (RKI). [Epidemiology of notifiable infectious diseases: 2011 annual report]. Berlin, Germany: RKI, 2012.
103 Robert Koch Insitut (RKI). [Epidemiology of notifiable infectious diseases: 2012 annual report]. Berlin, Germany: RKI, 2013.
104 Robert Koch Insitut (RKI). [Epidemiology of notifiable infectious diseases: 2013 annual report]. Berlin, Germany: RKI, 2014.
105 Robert Koch Insitut (RKI). [Epidemiology of notifiable infectious diseases: 2014 annual report]. Berlin, Germany: RKI, 2015.
106 Robert Koch Insitut (RKI). [Epidemiology of notifiable infectious diseases: 2015 annual report]. Berlin, Germany: RKI, 2016.
107 Robert Koch Insitut (RKI). [Epidemiology of notifiable infectious diseases: 2016 annual report]. Berlin, Germany: RKI, 2017.
108 Theano G. Meningococcal disease: epidemiological data in Greece (2004–2014). Hellenic Center for Disease Control & Prevention (HCDCP). Ministry of Health, 2016.
109 Health Protection Surveillance Centre (HPSC). Annual report 2010. Dublin, Ireland: Health Service Executive, 2011.
110 Health Protection Surveillance Centre (HPSC). Annual report 2011. Dublin, Ireland: Health Service Executive, 2012.
111 Health Protection Surveillance Centre (HPSC). Annual report 2012. Dublin, Ireland: Health Service Executive, 2013.
112 Health Protection Surveillance Centre (HPSC). Annual epidemiological report 2013. Dublin, Ireland: Health Service Executive, 2014.
113 Health Protection Surveillance Centre (HPSC). Annual epidemiological report 2014. Dublin, Ireland: Health Service Executive, 2015.
114 Health Protection Surveillance Centre (HPSC). Annual epidemiological report 2015. Dublin, Ireland: Health Service Executive, 2016.
115 Higher Institute of Health (ISS). [Surveillance data of invasive bacterial diseases updated to April 3, 2017]. Rome, Italy: ISS, 2017.
116 Egorova E, Otorbaeva D, Ronveaux O, Wasley A. Nationwide serogroup A meningococcal outbreak in Kyrgyzstan, 2014–2015. 13th Congress EMGM; 14–17 September 2015; Amsterdam, The Netherlands2015. p. 48.
117 Netherlands Reference Laboratory for Bacterial Meningitis. Bacterial meningitis in the Netherlands: annual report 2010. Amsterdam, The Netherlands: Academic Medicall Center (AMC) and National Institute of Public Health and the Environment (RIVM), 2011.
118 Netherlands Reference Laboratory for Bacterial Meningitis. Bacterial meningitis in the Netherlands: annual report 2011. Amsterdam, The Netherlands: Academic Medicall Center (AMC) and National Institute of Public Health and the Environment (RIVM), 2012.
119 Netherlands Reference Laboratory for Bacterial Meningitis. Bacterial meningitis in the Netherlands: annual report 2012. Amsterdam, The Netherlands: Academic Medicall Center (AMC) and National Institute of Public Health and the Environment (RIVM), 2013.
120 Netherlands Reference Laboratory for Bacterial Meningitis. Bacterial meningitis in the Netherlands: annual report 2013. Amsterdam, The Netherlands: Academic Medicall Center (AMC) and National Institute of Public Health and the Environment (RIVM), 2014.
121 Netherlands Reference Laboratory for Bacterial Meningitis. Bacterial meningitis in the Netherlands: annual report 2014. Amsterdam, The Netherlands: Academic Medicall Center (AMC) and National Institute of Public Health and the Environment (RIVM), 2015.
122 Netherlands Reference Laboratory for Bacterial Meningitis. Bacterial meningitis in the Netherlands: annual report 2015. Amsterdam, The Netherlands: Academic Medicall Center (AMC) and National Institute of Public Health and the Environment (RIVM), 2016.

123 Van Der Ende A, Arends A, Feller M, Keijzers W, Schurman I, Knol M, et al. Invasive meningococcal disease in the Netherlands, 2015–2016. 14th Congress EMGM; 18–21 September 2017; Prague, Czech Republic2017. p. 70.
124 Blystad H, Caugant DA, Haugen IL, Rønning K, Steens A, Steinbakk M, et al. [Disease program annual report 2011: invasive infections]. Oslo, Norway: Public Health Institute Division of Infectious Disease, 2012.
125 Blystad H, Caugant DA, Haugen IL, Konsmo K, Steens A, Steinbakk M, et al. [Disease program annual report 2012: invasive infections]. Oslo, Norway: Public Health Institute Division of Infectious Disease, 2013.
126 Blystad H, Caugant DA, Haugen IL, Konsmo K, Steens A, Steinbakk M, et al. [Disease program annual report 2013: invasive infections]. Oslo, Norway: Public Health Institute Division of Infectious Disease, 2014.
127 Caugant DA, Haugen IL, Konsmo K, Nordstrand K, Steinbakk M, Storsæter J, et al. [Disease program annual report 2014: invasive infections]. Oslo, Norway: Public Health Institute Division of Infectious Disease, 2015.
128 Caugant DA, Haugen IL, Konsmo K, Nordstrand K, Steinbakk M, Storsæter J, et al. [Disease program annual report 2015: invasive infections]. Oslo, Norway: Public Health Institute Division of Infectious Disease, 2016.
129 Berg A, Caugant DA, Haugen IL, Konsmo K, Steinbakk M, Storsæter J, et al. [Disease program annual report 2016: invasive infections]. Oslo, Norway: Public Health Institute Division of Infectious Disease, 2017.
130 Skoczyńska A, Kuch A, Waśko I, Gołębiewska A, Ronkiewicz P, Markowska M, et al. [Invasive meningococcal disease in patients under 20 years of age in Poland 2009–2011]. Pediatria Polska. 2012;87:438–43.
131 Waśko I, Kuch A, Kiedrowska M, Gołębiewska A, Ronkiewicz P, Wróbel I, et al. Invasive meningococcal disease in Poland, 2015–2016. 14th Congress EMGM; 18–21 September 2017; Prague, Czech Republic2017. p. 61.
132 Simões MJ, Fernandes T. [Invasive meningococcal disease in Portugal: integrated epidemiologic surveillance, 2003–2014]. Lisbon, Portugal: National Institute of Health (INSA, IP), 2016.
133 Institutul National de Sanatate Publica (INSP). [Analysis of the evolution of communicable diseases under surveillance: annual report for 2010]. INSP, 2011 ISNN 2537-2394.
134 Institutul National de Sanatate Publica (INSP). [Analysis of the evolution of communicable diseases under surveillance: annual report for 2015]. INSP, 2016.
135 Koroleva I, Beloshitskij G, Zakroeva I, Melnikova A, Koroleva M, Shipulin G, et al. Invasive meningococcal disease in Russian Federation. The European Meningococcal Disease Society: 20 Years EMGM; 17–19 September 2013; Bad Loipersdorf, Austria2013. p. 71.
136 Koroleva I, Beloshitskiy I, Zakroeva I, Koroleva M. Invasive meningococcal disease in Russian Federation. 13th Congress EMGM; 14–17 September 2015; Amsterdam, The Netherlands2015. p. 51.
137 Koroleva I, Melnikova A, Koroleva M. Invasive meningococcal disease in Russian Federation. 14th Congress EMGM; 18–21 September 2017; Prague, Czech Republic2017. p. 100.
138 Kruzlíková A. Invasive meningococcal diseases and aspect of nasofaryngeal state carrier in Slovakia in a year 2015 and 2016. 14th Congress EMGM; 18–21 September 2017; Prague, Czech Republic2017. p. 111.
139 Garrido Estepa M, Manguiña Guzmán M, Cano Portero R. [Meningococcal Disease in Spain: analysis of the 2012–2013 time period]. National Center of Epidemiology: Carlos III Health Institute (ISCIII), 2014.
140 National Center of Epidemiology: Health Institute Carlos III (ISCIII). [Weekly surveillance report: October 6, 2015]. ISCIII, 2015.
141 National Center of Epidemiology: Health Institute Carlos III (ISCIII). [Weekly surveillance report: August 23, 2016]. ISCIII, 2016.
142 Public Health Agency Sweden. Invasive meningococcal infection. 2017. Available: https://www.folkhalsomyndigheten.se/folkhalsorapportering-statistik/statistikdatabaser-och-visualisering/sjukdomsstatistik/meningokockinfektion-invasiv/?t=com&p=5809. Accessed.
143 Ninet B, Schrenzel J. Annual report of the National Center for Meningococci 2010. Geneva, Switzerland: National Center for Meningococci (NCM), 2011.
144 Emonet S, Schrenzel J. Annual Report of the National Center for Meningococci 2011. Geneva, Switzerland: National Center for Meningococci (NCM), 2012.
145 Hinrikson H, Emonet S, Schrenzel J. Annual report of the National Center for Meningococci 2012. Geneva, Switzerland: Swiss National Center for Meningococci (CNM), 2013.
146 Hinrikson H, Emonet S, Schrenzel J. Swiss National Reference Center for Meningococci: 2013 annual report. Geneva, Switzerland: Swiss National Reference Center for Meningococci (CNM), 2014.
147 Hinrikson H, Emonet S, Schrenzel J. Swiss National Reference Center for Meningococci: 2014 annual report. Geneva, Switzerland: Swiss National Reference Center for Meningococci (CNM), 2015.
148 Hinrikson H, Emonet S, Schrenzel J. Swiss National Reference Center for Meningococci: 2015 annual report. Geneva, Switzerland: Swiss National Reference Center for Meningococci (CNM), 2016.

149 Ceyhan M, Gürler N, Ozsurekci Y, Keser M, Aycan AE, Gurbuz V, et al. Meningitis caused by *Neisseria meningitidis*, *Hemophilus influenzae* type B and *Streptococcus pneumoniae* during 2005–2012 in Turkey: a multicenter prospective surveillance study. Human Vaccines & Immunotherapeutics. 2014;10:2706–12.
150 Ceyhan M, Ozsurekci Y, Gürler N, Karadag Oncel E, Camcioglu Y, Salman N, et al. Bacterial agents causing meningitis during 2013–2014 in Turkey: A multi-center hospital-based prospective surveillance study. Human Vaccines & Immunotherapeutics. 2016;12:2940–5.
151 Ladhani SN, Flood JS, Ramsay ME, Campbell H, Gray SJ, Kaczmarski EB, et al. Invasive meningococcal disease in England and Wales: implications for the introduction of new vaccines. Vaccine. 2012;30:3710–6.
152 Public Health England. Invasive meningococcal infections (England and Wales), annual report for 2011/12. Health Protection Report. 2013;7.
153 Public Health England. Laboratory confirmed reports of invasive meningococcal infections in England: 2012/2013 annual data by epidemiological year. Health Protection Report. 2014;8:7–10.
154 Public Health England. Invasive meningococcal disease (laboratory reports in England): 2013/2014 annual data by epidemiological year. Health Protection Report. 2015;9.
155 Public Health England. Invasive meningococcal disease (laboratory reports in England): 2015/2016 annual data by epidemiological year. Health Protection Report. 2016;10.
156 Public Health England. Laboratory confirmed reports of invasive meningococcal disease in England: July to September 2016. Health Protection Report. 2016.
157 Public Health England. Laboratory confirmed reports of invasive meningococcal disease in England: October to December 2016. Health Protection Report. 2017.
158 Public Health Wales. Meningitis and meningococcal disease. 2017. Available: http://www.wales.nhs.uk/sites3/page.cfm?orgId=457&pid=32261#2015. Accessed.
159 Public Health Agency (PHA). Meningococcal disease in Northern Ireland. Belfast, Northern Ireland, UK: PHA, 2017.
160 McDonald E, Denham B, McMenamin J, Cameron C. Respiratory and immunisation quarterly report, quarter four: 1 October to 31 December 2010. HPS Weekly Report. 2011;45:95–8.
161 McDonald E, Denham B, Smith-Palmer A, McMenamin J. Respiratory bacteria quarterly report, quarter four: 1 October to 31 December 2011. HPS Weekly Report. 2012;46:81–3.
162 Wissmann BV, Denham B, Smith-Palmer A, Cameron C. Respiratory bacteria quarterly report, quarter four: 1 October to 31 December 2012. HPS Weekly Report. 2013;47:74–6.
163 The Vaccine Preventable Diseases Team and the Scottish *Haemophilus Legionella* Meningococcus and Pneumococcus Reference Laboratory (SHLMPRL). Respiratory bacteria quarterly report, quarter four: 1 October to 31 December 2013. HPS Weekly Report. 2014;48:120–6.
164 The Vaccine Preventable Diseases Team and the Scottish *Haemophilus Legionella* Meningococcus and Pneumococcus Reference Laboratory (SHLMPRL). Respiratory bacteria quarterly report, quarter four: 1 October to 31 December 2014. HPS Weekly Report. 2015;49:90–7.
165 The Vaccine Preventable Diseases Team and the Scottish *Haemophilus Legionella* Meningococcus and Pneumococcus Reference Laboratory (SHLMPRL). Respiratory bacteria quarterly report, quarter four: 1 October to 31 December 2015. HPS Weekly Report. 2016;50:87–94.
166 Health Protection Scotland Immunisation team and the Scottish *Haemophilus Legionella* Meningococcus and Pneumococcus Reference Laboratory (SHLMPRL). Respiratory bacteria quarterly report, quarter three: 1 July to 31 September 2016. HPS Weekly Report. 2016;50:425–33.
167 European Centre for Disease Prevention and Control (ECDC). Surveillance atlas of infectious diseases. 2017. Available: http://ecdc.europa.eu/en/data-tools/atlas/Pages/atlas.aspx. Accessed.
168 Majumdar T, Bhattacharya S, Barman D, Baidya S. Impact of vaccination following outbreak of meningococcal infection in Tripura, India. Journal of Pharmaceutical and Biomedical Sciences. 2013;2013:1853–59.
169 NNDSS Annual Report Writing Group. Australia's notifiable disease status, 2010: annual report of the National Notifiable Diseases Surveillance System Communicable Diseases Intelligence. 2012;36:1–69.
170 Lahra MM, Enriquez RP. Annual report of the Australian Meningococcal Surveillance Programme, 2011. Communicable Diseases Intelligence. 2012;36:E251–E62.
171 Lahra MM, Enriquez RP. Australian Meningococcal Surveillance Programme annual report, 2012. Communicable diseases intelligence quarterly report. 2013;37:E224–E32.
172 Lahra MM, Enriquez RP. Australian Meningococcal Surveillance Programme annual report, 2013. Communicable Diseases Intelligence. 2014;38:E301–E8.
173 Lahra MM, Enriquez RP. Australian Meningococcal Surveillance Programme annual report, 2014. Communicable Diseases Intelligence. 2016;40:E221–E8.

174 Lahra MM, Enriquez RP, National Neisseria Network. Australian Meningococcal Surveillance Programme annual report, 2015. Communicable Diseases Intelligence Quarterly Report. 2016;40:E503–E11.
175 Lahra MM, Enriquez RP. Australian Meningococcal Surveillance Programme, 1 July to 30 September 2016. Communicable Diseases Intelligence Quarterly Report. 2016;40:E560.
176 Li J-h, Li Y-x, Wu D, Ning G-j, Shao Z-J, Yin Z-d. [Epidemiological characteristics of meningococcal meningitis and switching trend of serogroups of *Neisseria meningitidis* in China, 2006–2014]. Chinese Journal of Vaccines and Immunization. 2015;21:481–5.
177 National Institute of Infectious Diseases (NIID). [Trends in invasive meningococcal disease, week 13 2013 to week 52 2014, Japan]. Infectious agents surveillance report (IASR). 2015;36:179–81.
178 Lopez L, Sexton K, Carter P. The epidemiology of meningococcal disease in New Zealand in 2010. Wellington, New Zealand: Institute of Environmental Science and Research Ltd (ESR), 2011.
179 Lopez L, Sexton K, Carter P. The epidemiology of meningococcal disease in New Zealand in 2011. Wellington, New Zealand: Institute of Environmental Science and Research Ltd (ESR), 2012.
180 Lopez L, Sexton K. The epidemiology of meningococcal disease in New Zealand in 2012. Wellington, New Zealand: Institute of Environmental Science and Research Ltd (ESR), 2013.
181 Lopez L, Sherwood J. The epidemiology of meningococcal disease in New Zealand in 2013. Wellington, New Zealand: Institute of Environmental Science and Research Ltd (ESR), 2014.
182 Institute of Environmental Science and Research Ltd (ESR). Notifiable diseases in New Zealand: annual report 2014. Porirua, New Zealand: ESR, 2015.
183 Institute of Environmental Science and Research Ltd (ESR). Notifiable diseases in New Zealand: annual report 2015. Porirua, New Zealand: ESR, 2016.
184 Institute of Environmental Science and Research Ltd (ESR). Notifiable diseases in New Zealand: annual report 2016. Porirua, New Zealand: ESR, 2017.
185 Réseau Algérien de Surveillance de la Résistance des Bactéries aux Antibiotiques (AARN). [Bacterial antimicrobial resistance surveillance: 14th Evaluation Report]. Ministère de la Santé, de la Population et de la Réforme Hospitalière, République Algérienne Démocratique et Populaire, 2015.
186 Réseau Algérien de Surveillance de la Résistance des Bactéries aux Antibiotiques (AARN). [Bacterial antimicrobial resistance surveillance: 15th Evaluation Report]. Ministère de la Santé, de la Population et de la Réforme Hospitalière, République Algérienne Démocratique et Populaire, 2016.
187 Nambei WS, Gamba EP, Gbangbangai E, Ouambita RM, Dalengat-Vogbia Z, Nana R, et al. Detection of the serogoups and serotypes causing bacterial meningitidis in Bangui, 2012. Medecine et Sante Tropicales. 2016;26:302–7.
188 Dalecha D. Meningitis outbreak investigation-Kembata zone, Southern Ethiopia, February, 2011. International Journal of Infectious Diseases. 2012;16:e252.
189 Traore FA, Sako FB, Sylla D, Kader DS, Bangoura M, Traore M, et al. [Epidemic meningitis in 2013 in Republic of Guinea: Nesseria meningitidis W135 emergence]. Bulletin de la Societe de Pathologie Exotique. 2016;109:364-7.
190 Caugant DA, Kristiansen PA, Wang X, Mayer LW, Taha MK, Ouedraogo R, et al. Molecular Characterization of Invasive Meningococcal Isolates from Countries in the African Meningitis Belt before Introduction of a Serogroup A Conjugate Vaccine. Plos One. 2012;7.
191 Tsang RSW, Law DKS, Gad RR, Mailman T, German G, Needle R. Characterization of invasive Neisseria meningitidis from Atlantic Canada, 2009 to 2013: With special reference to the nonpolysaccharide vaccine targets (PorA, factor H binding protein, Neisseria heparin-binding antigen and Neisseria adhesin A). Canadian Journal of Infectious Diseases & Medical Microbiology. 2015;26:299-304.
192 Chanto G. [Laboratory surveillance report: *Neisseria meningitidis*, Costa Rica, 2006–2015]. Tres Ríos, Costa Rica: Instituto Constarricense de Investigación y Enseñanza en Nutrición y Salud (INCIENSA),, 2016.
193 Chacon-Cruz E, Martinez-Longoria CA, Llausas-Magana E, Luevanos-Velazquez A, Vazquez-Narvaez JA, Beltran S, et al. *Neisseria meningitidis* and *Streptococcus pneumoniae* as leading causes of pediatric bacterial meningitis in nine Mexican hospitals following 3 years of active surveillance. Therapeutic Advances in Vaccines. 2016;4:15–9.
194 Cabrera-Gaytan DA, Perez-Perez GF, Arriaga-Nieto L, Vallejos-Paras A, Padilla-Velazquez R, Grajales-Muniz C. Epidemiological surveillance of meningeal and encephalic syndrome in the Mexican social security Institute, 2012–2014. Revista Medica del Hospital General de Mexico. 2017;80:130–8.
195 Husain EH, Barakat M, Al-Saleh M. Trends and variations in the epidemiology of meningococcal disease in Kuwait 1987-2013. Journal of Infection and Public Health. 2015;8:441-7.

196 Bukovski S, Vacca P, Anselmo A, Knezovic I, Fazio C, Neri A, et al. Molecular characterization of a collection of Neisseria meningitidis isolates from Croatia, June 2009 to January 2014. Journal of Medical Microbiology. 2016;65:1013–9.

197 Klismanic Z, Juretic KB, Tripkovic I. Epidemiological characteristics of invasive meningococcal disease in the Split-Dalmatia County 1996-2010. Acta clinica Croatica. 2013;52:485-91.

198 Bukovski S, Švagelj A, Kaić B. Invasive disease caused by *Neiseria meningitidis*, Croatia January 2015–May 2017. 14th Congress EMGM; 18–21 September 2017; Prague, Czech Republic2017. p. 76.

199 Eloshvili M, McHedlishvili I, Imnadze P. [Epidemiology of meningococcal infection in Georgia]. Georgian Medical News. 2012:37-40.

200 Infectious Disease Prevention and Control Unit. Communicable disease control strategy for Malta: 2013. Health Promotion and Disease Prevention Directorate, 2013.

201 National Reference Laboratory for Meningococcus and Haemophilus (NRLMH). [National Reference Laboratory for Meningococcus and Haemophilus]. Public Health Institute Sombor, 2015.

202 National Institute of Infectious Diseases (NIID). Meningococcal disease cases in Scotland and Sweden, following attendance at the World Scout Jamboree, Yamaguchi, Japan, July 28–August 8, 2015. 2015.
